# Supplementary material for: Comprehensive analysis of Saccharomyces cerevisiae intron structures in vivo
Source: Nat Struct Mol Biol. 2025 Jun 5;32(8):1488–502. doi: 10.1038/s41594-025-01565-x (PMC12350175; doi:10.1038/s41594-025-01565-x)
Supplement: Supplementary file 1 — Supplementary Text, Supplementary Figs. 1–20, Supplementary Table 2 and Source data for Supplementary Fig. 13. [file 41594_2025_1565_MOESM1_ESM.pdf]

nature structural & molecular biology

Supplementary information https://doi.org/10.1038/s41594-025-01565-x

Comprehensive analysis of *Saccharomyces cerevisiae* intron structures in vivo

In the format provided by the authors and unedited

Supplemental Text

Alternative DMS-guided structure prediction approaches. We explored additional structure prediction approaches to identify potential pseudoknots and alternative conformations in introns using the DMS data. To find potential pseudoknots, structural motifs that often participate in stable three-dimensional RNA folds, we predicted structures using ShapeKnots[1] guided by DMS data for each intron. No introns included predicted pseudoknots with helix confidence estimates exceeding the 70% confidence threshold. As a control for our pseudoknot predictions, we predicted the secondary structure for the RNase P RNA with ShapeKnots[1] guided by DMS data. The known pseudoknot in RNAse P[2] was recovered with this approach but only with 21% helix confidence estimate, suggesting that some pseudoknots in introns may be missed in our scan due to low sensitivity when using the 70% helix confidence threshold.

Additionally, for eight intron regions with high coverage (see Methods), we generated structure predictions using DREEM[3] to test for alternate conformations represented by the DMS data. However, reactivity data for all eight regions were best explained by a single structure based on the Bayesian Information Criteria (BIC) test statistic reported by DREEM. We note that it is possible these introns have significant alternative conformations that would only become apparent when probing with higher DMS concentrations or sequencing more deeply.

Detailed evaluation of proposed functional structures in *S. cerevisiae* introns. Our DMS-MaPseq data after splicing inhibition allowed us to assess classes of previously proposed intron structures in *S. cerevisiae*. These classes include intron structures that were identified through mutational studies and functional experiments, along with introns identified through computational structure prediction and evolutionary analysis. We find that structures identified with functional experiments or scans for covariation were largely supported by our *in vivo* chemical probing data, whereas structures identified through other computational prediction approaches had limited support. Here, we include a detailed description of these classes of structures along with their support from our DMS data.

Prior studies have used experiments assessing the role of structure using mutants and compensatory mutants to pinpoint to potential regulatory structures in some *S. cerevisiae* introns. For instance, in the case of *RPS17B*, a stem linking the 5' splice site to the branch point, termed a "zipper stem", enables efficient splicing despite this intron's weak 5' splice site.[4,5] A stem between the branch point and 3' splice site in RPS23B hides a more proximal cryptic 3' splice site and enables thermosensitive regulation of 3' splice site selection for this intron.[6] In the case of the introns in *RPL32*, *RPS9A*, and *RPS14B*, portions of the intron have been implicated in regulating gene expression by binding of excess protein product to pre-mRNA, leading to gene-specific reduced splicing efficiency.[7-10] Finally, structures in introns from *RPL18A* and *RPS22B* have been found to mediate the degradation of unspliced pre-mRNA.[11]

In four out of six cases, structures from prior functional experiments received medium or high support from our DMS data, with high loop reactivity, low stem reactivity, and high helix confidence estimates (Fig. 2). For instance, the structure in RPL18A involved in pre-mRNA degradation includes high confidence stems from DMS probing (Fig. 2A). DMS data additionally support the intron stem in *RPS23B* co-localizing the branch point and 3' splice site (Fig. 2B), and high confidence stems are identified in the secondary structure proposed in

RPS14B to bind the RPS14B protein (Fig. 2C). Finally, high confidence stems are predicted in the *RPS9A* intron structure proposed to regulate gene expression levels of *RPS9A* and *RPS9B* (Fig. 2E). In other cases, proposed structures are not supported by the probing data, with DMS accessible residues present in these structures' stems. These lower support structures include the zipper stem in the *RPS17B* intron (Supplementary Fig. 5A), and the *RPL30* structure proposed to regulate gene expression (Supplementary Fig. 5B). The structure in *RPS22B* found to trigger pre-mRNA degradation was not assessed, as this intron did not pass our DMS-MaPseq coverage threshold. With most structures in this class showing medium or high DMS support, proposed structures from functional experiments assessing mutants and compensatory mutants are in general validated by our data (Fig. 2G).

We next evaluated structures identified by Hooks, et al. (2016)[12] through predictions from CMfinder[13], RNAz[14], and Evofold[15], approaches that use sequence alignments to identify structures. These structures showed mixed support from DMS data, with introns in *YRA1*, *RPL18B*, *RPL7A*, and *RPL28* including some stems with high confidence and other stems with low DMS support (Supplementary Fig. 6A-D). The intron structures in *RPL22B* and *GLC7* were not predicted from the DMS data (Supplementary Fig. 6E-F). Proposed intron structures in *MPT5* and *RPS22B* could not be evaluated due to low sequencing coverage. Unlike R-scape, RNAz uses thermodynamics calculations and conservation alone rather than covariation, and CMfinder and Evofold do not use phylogeny sequence backgrounds to identify significant covariation. With a higher fraction of predicted structures that were not validated by DMS data from this set (Fig. 2G), these approaches appear to less reliably identify structures that form *in vivo* compared to functional experiments.

We evaluated intron structures that included covarying residues using DMS data, finding that in the majority of cases, covarying base-pairs were supported by the DMS data. Six of the seven snoRNA-containing introns exhibited sufficient coverage from DMS-MaPseq for evaluation. In these six introns, a majority of covarying residues were supported by DMS data, with 20 of the 27 covarying residues present in high-confidence stems from DMS-guided structure prediction (Supplementary Fig. 8). Beyond snoRNA-containing introns, covarying residues were identified in four introns from our covariation scan and in two introns from Gao, et al. (2021).[16] First, we identified covariation in stems of *RPL7A*'s first intron, and these covarying residues were included in high confidence stems forming a 3-way junction (Fig. 2D). Next, as noted by sequence analysis in Plocik and Guthrie (2012),[9] we found signals for covariation in a hairpin shared by the introns in *RPS9A* and *RPS9B*, and covarying base pairs in these two introns align with the secondary structure from DMS-MaPseq (Fig. 2E-F). Only the structure in *RPS13* noted to have covarying residues in Gao, et al. (2021)[16] was assigned low helix confidence estimates (22%), suggesting that these residues are not paired in the dominant *in vivo* structure (Supplementary Fig. 5C). The first intron in *RPL7B*, found to have covarying residues by Gao, et al. (2021)[16] and in our covariation scan, did not result in enough sequencing coverage from DMS-MaPseq for analysis. Overall, the prevalence of DMS-validated covarying residues across these cases suggests that covariation from R-scape[17] can reliably identify structures that form *in vivo* (Fig. 2G).

Intron RNA folding with *in vitro* M2-seq. Our DMS-guided intron secondary structure predictions suggested that *in vivo*, *S. cerevisiae* introns harbor extended secondary structure, with longer, high-confidence stems compared to coding regions. We explored the structure of introns

outside the nuclear environment through *in vitro* structure probing. Here we discuss in detail our findings from *in vitro* probing with M2-seq for a set of *in vitro* transcribed candidate introns.

To assess the *in vitro* folding of individual intron RNAs, we turned to mutate-and-map readout through next-generation sequencing (M2-seq[18]) on introns that were *in vitro* transcribed and probed separate from other RNA. M2-seq allowed us to assess the formation of base-pairs found from DMS-guided secondary structure prediction *in vitro*, identifying base-pairing partners in addition to providing average per-residue accessibility data. For each intron, we generated a pool of RNA *in vitro* transcribed with sparse errors that were installed via error-prone PCR. These mutations can lead RNA molecules to adopt altered secondary structure ensembles, with a mutated residue in a stem exposing its base-pairing partner in solution and potentially leading to unfolding of the stem. Chemical probing of this RNA pool by DMS, in addition to modifying positions that are accessible in the wildtype RNA, also modifies base-pairing partners for mutated stem residues and any other newly accessible positions in the mutated RNA. Off-diagonal signals in the resulting background-subtracted Z-score plots indicate the presence of a stem, as these appear when one residue's mutation leads to an increase in another residue's accessibility. In the case of the introns in *QCR9* and *RPL36B*, the Z-score plots from *in vitro* M2-seq included these off-diagonal signals, with mutations in some positions resulting in increased DMS reactivity at other distal positions (Fig. 4A, Extended Data Fig. 6A). When using RNAstructure to predict secondary structures for these constructs using both 1D and 2D reactivity data, the resulting helix confidence estimates from bootstrapping confirm the formation of stable stems in both cases (Fig. 4C, Extended Data Fig. 6C). The Z-scores include two-dimensional reactivity signals for many of these stems (Fig. 4A, Extended Data Fig. 6A), and the resulting base-pairing probabilities support the formation of these stems as these introns' primary structure (Fig. 4B, Extended Data Fig. 6B). These secondary structures agree with the stems observed *in vivo*, with high confidence stems *in vivo* also appearing in the *in vitro* M2-seq structures (Fig. 4C-D, Extended Data Fig. 6C-D).

VARS-seq experimental design and validation. To evaluate the effects of intron structure variants with VARS-seq, we integrated genes containing intron libraries into the yeast genome[19] (Fig. 6A). Reasoning that exogenous reporter genes can alter pre-mRNA secondary structure and splicing patterns, we instead integrated them into the genome in their full native gene context with CRISPR/Cas9 (Extended Data Fig. 8C). Random barcodes (12N) were installed in the 5′ UTR upstream of the intron to serve two roles. First, these sequences helped link spliced reads to the pre-mRNA variant they originated from. Second, these randomized sequences provided perturbations for the efficiency of splicing and mRNA decay; these perturbations are especially useful to identify effects in cases where wildtype retained intron levels are beyond the dynamic range for our assay. For instance, in the case that retained intron levels are low with highly efficient splicing for a wildtype intron, this barcoding strategy can provide additional dynamic range for our assay because some randomly designed barcode sequences will attenuate splicing or pre-mRNA decay rates. In particular, by measuring the effects of many unique barcode sequences per intron variant, we can observe the distribution of retained intron levels for wildtype and variant sequences across barcodes, robustly identifying variants that shift this distribution and alter gene expression.

We sequenced genomic DNA (gDNA) to link randomized barcodes to intron variant sequences and obtain transformation frequencies, and we measured spliced and unspliced RNA levels with

targeted RNA sequencing (Fig. 6A). Reassuringly, most consensus variant sequences obtained from gDNA sequencing were the expected library length (between 71.5% and 84.8% across sub-libraries, Supplementary Fig. 13A), and most designed variants were assigned to at least 10 unique barcodes (92.1%, Supplementary Fig. 13B). Our targeted RNA sequencing measurements were free from genomic DNA contamination (Supplementary Fig. 13C), and as expected, variants that disrupted key splice site sequences (branch point mutants in QCR9) significantly increased retain intron levels (Supplementary Fig. 13D). Though we did observe alternative splicing events from our RNA sequencing data, all 6 observed events represented a minor population of transcripts produced from variants including these events (Supplementary Fig. 13E). Using our DNA and RNA sequencing data, we computed the following two metrics for each barcode and variant sequence: the retained intron (RI) fraction (fraction of RNA-seq reads that were unspliced) and the normalized mRNA level (spliced mRNA read counts normalized by the gDNA read counts). Though many variants led to small changes in these two readouts compared to wildtype sequences, barcode sequences enabled us to robustly identify even subtle effects by providing perturbations that altered baseline splicing and decay rates.

Comparing intron DMS-guided structure prediction with *de novo* structure prediction. We found that many structural features that were enriched in introns when using DMS-guided structure prediction remained enriched when using *de novo* structure prediction. Zipper stems and downstream stems in introns are more stable (lower dG values) than those in control sequences, whether using DMS-guided structure prediction, *de novo* minimum-free energy structure prediction, or *de novo* secondary structure ensemble prediction (p-values < 0.01, blue and orange in Fig. 7B). Introns have longer stems than length-matched control sequences and have higher maximum extrusion from ends, again both from *de novo* structure predictions and DMS-guided structure predictions (p-values < 0.01, green and red in Fig. 7B). We additionally measured the secondary structure graph distance between the 5' splice site and branch point sequence positions, comparing between introns and the control sequences (purple in Fig. 7B). From DMS-guided structures, the 5' splice site and branch point were more distant in introns than controls (p-value < 0.01), perhaps due to the requirement for single-stranded nucleotides proximal to these sequences in the A-complex spliceosome. On the other hand, *de novo* structure prediction suggests that introns have lower secondary structure graph distances between the 5' splice site and branch point without the context of the spliceosome (p-values < 0.01), as found by Rogic, et al. (2008),[5] suggesting that introns co-localize key splicing sequences. We tested other structure prediction approaches and found similar structural feature enrichment, comparing against other control sets, using an alternate folding package, and making predictions including exonic context (Supplementary Fig. S18). In particular, the enrichment for stable zipper stems, higher maximum extrusion from ends, and lower 5' splice site to branch point distances remained significant across all tested *de novo* prediction approaches.

References

1. Hajdin, C.E. et al. Accurate SHAPE-directed RNA secondary structure modeling, including pseudoknots. *Proc Natl Acad Sci U S A* **110**, 5498-503 (2013).
2. Lan, P. et al. Structural insight into precursor tRNA processing by yeast ribonuclease P. *Science* **362**(2018).

3. Tomezsko, P.J. et al. Determination of RNA structural diversity and its role in HIV-1 RNA splicing. *Nature* **582**, 438-442 (2020).
4. Charpentier, B. & Rosbash, M. Intramolecular structure in yeast introns aids early steps of in vitro spliceosome assembly. *RNA* (1996).
5. Rogic, S. et al. Correlation between the secondary structure of pre-mRNA introns and the efficiency of splicing in Saccharomyces cerevisiae. *BMC Genomics* **9**, 355 (2008).
6. Meyer, M., Plass, M., Perez-Valle, J., Eyras, E. & Vilardell, J. Deciphering 3'ss selection in the yeast genome reveals an RNA thermosensor that mediates alternative splicing. *Mol Cell* **43**, 1033-9 (2011).
7. Vilardell, J. & Warner, J.R. Regulation of splicing at an intermediate step in the formation of the spliceosome. *Genes Dev* **8**, 211-20 (1994).
8. Li, Z., Paulovich, A.G. & Woolford, J.L., Jr. Feedback inhibition of the yeast ribosomal protein gene CRY2 is mediated by the nucleotide sequence and secondary structure of CRY2 pre-mRNA. *Mol Cell Biol* **15**, 6454-64 (1995).
9. Plocik, A.M. & Guthrie, C. Diverse forms of RPS9 splicing are part of an evolving autoregulatory circuit. *PLoS Genet* **8**, e1002620 (2012).
10. Fewell, S.W. & Woolford, J.L., Jr. Ribosomal protein S14 of Saccharomyces cerevisiae regulates its expression by binding to RPS14B pre-mRNA and to 18S rRNA. *Mol Cell Biol* **19**, 826-34 (1999).
11. Danin-Kreiselman, M., Lee, C.Y. & Chanfreau, G. RNAse III-Mediated Degradation of Unspliced Pre-mRNAs and Lariat Introns. *Molecular Cell* **11**, 1279-1289 (2003).
12. Hooks, K.B., Naseeb, S., Parker, S., Griffiths-Jones, S. & Delneri, D. Novel Intronic RNA Structures Contribute to Maintenance of Phenotype in Saccharomyces cerevisiae. *Genetics* **203**, 1469-81 (2016).
13. Yao, Z., Weinberg, Z. & Ruzzo, W.L. CMfinder--a covariance model based RNA motif finding algorithm. *Bioinformatics* **22**, 445-52 (2006).
14. Gruber, A.R., Neubock, R., Hofacker, I.L. & Washietl, S. The RNAz web server: prediction of thermodynamically stable and evolutionarily conserved RNA structures. *Nucleic Acids Res* **35**, W335-8 (2007).
15. Pedersen, J.S. et al. Identification and classification of conserved RNA secondary structures in the human genome. *PLoS Comput Biol* **2**, e33 (2006).
16. Gao, W., Jones, T.A. & Rivas, E. Discovery of 17 conserved structural RNAs in fungi. *Nucleic Acids Res* **49**, 6128-6143 (2021).
17. Rivas, E., Clements, J. & Eddy, S.R. A statistical test for conserved RNA structure shows lack of evidence for structure in lncRNAs. *Nat Methods* **14**, 45-48 (2017).
18. Cheng, C.Y., Kladwang, W., Yesselman, J.D. & Das, R. RNA structure inference through chemical mapping after accidental or intentional mutations. *Proc Natl Acad Sci U S A* **114**, 9876-9881 (2017).
19. Lee, M.E., DeLoache, W.C., Cervantes, B. & Dueber, J.E. A Highly Characterized Yeast Toolkit for Modular, Multipart Assembly. *ACS Synth Biol* **4**, 975-86 (2015).

Supplemental Figures

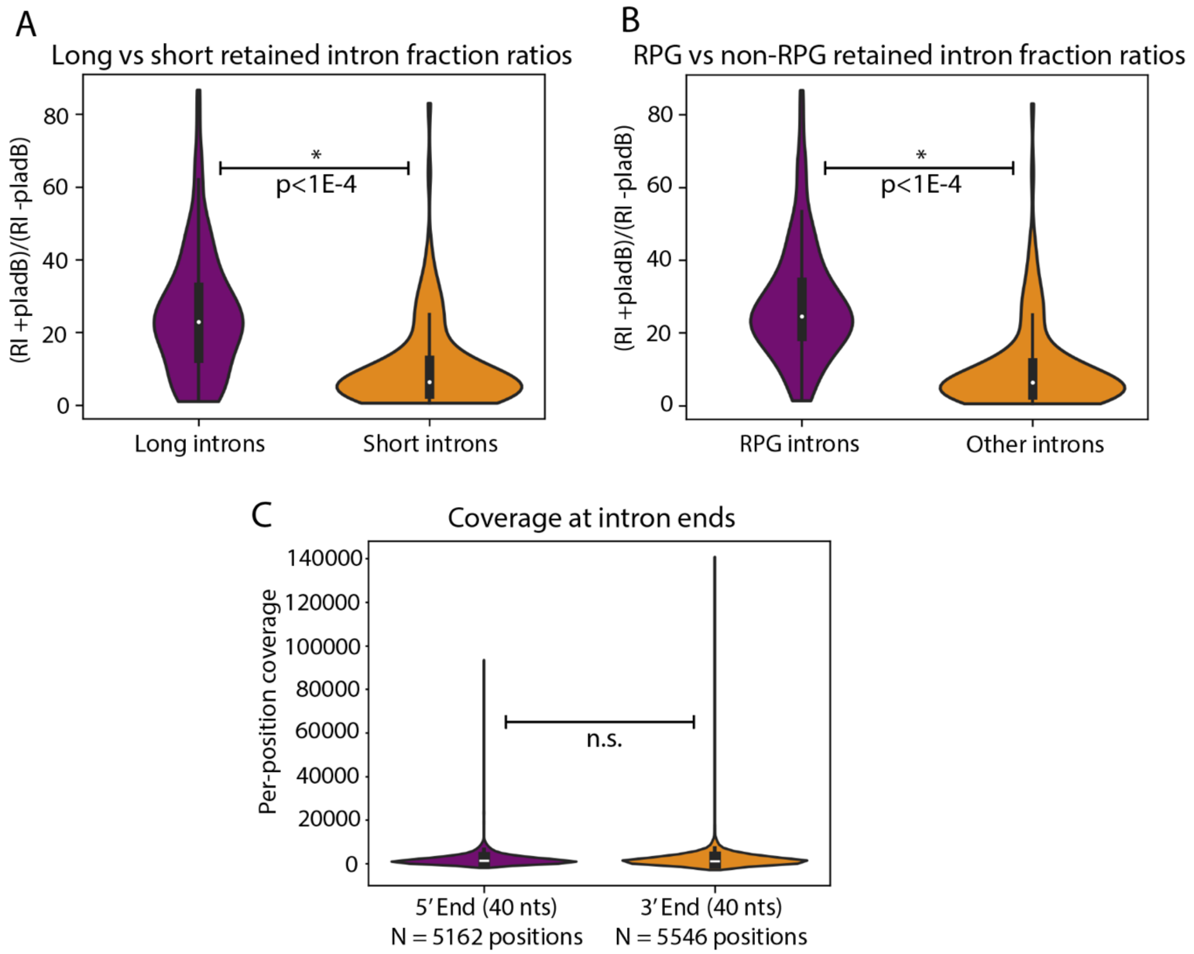

Supplementary Figure 1: Analyzing effects of pladB treatment on intron retention and intron degradation. Comparing the ratio of the retained intron (RI) fraction between **A)** 123 long (> 200 nucleotides) and 118 short (< 200 nucleotides) introns, and between **B)** 103 introns in ribosomal protein coding genes (RPGs) vs 138 other introns. **C)** Comparing per-position coverage at the 5' end and 3' end of all introns after pladB treatment (no significant difference at p=0.05 threshold). p-values were computed using two-sided Wilcoxon ranked sum tests to compare classes. Box plots mark the median as the center white point and include a box from the 25th (Q1) to 75th (Q3) percentile, extending whiskers to the smallest and largest value that fall within 1.5 times the interquartile range below Q1 and above Q3.

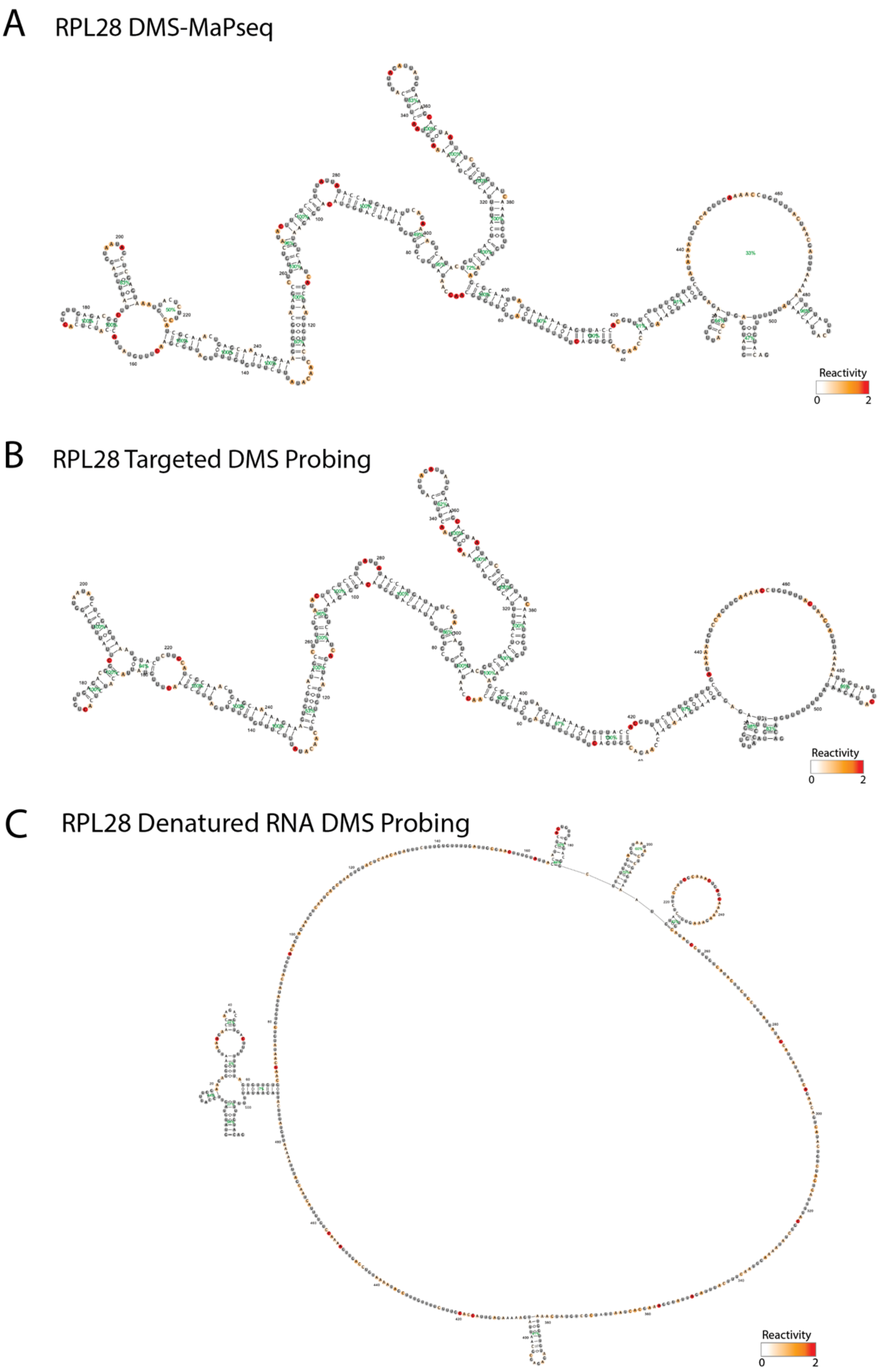
A RPL28 DMS-MaPseq
Reactivity
0
2
B RPL28 Targeted DMS Probing
Reactivity
0
2
C RPL28 Denatured RNA DMS Probing
Reactivity
0
2

D RPL7A DMS-MaPseq

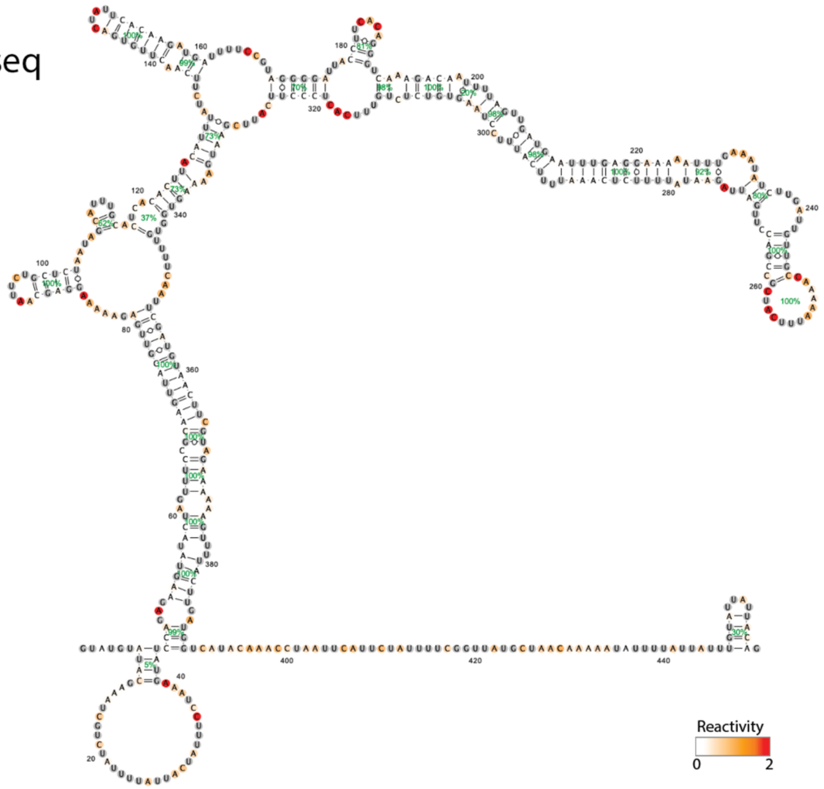

E RPL7A Targeted DMS Probing

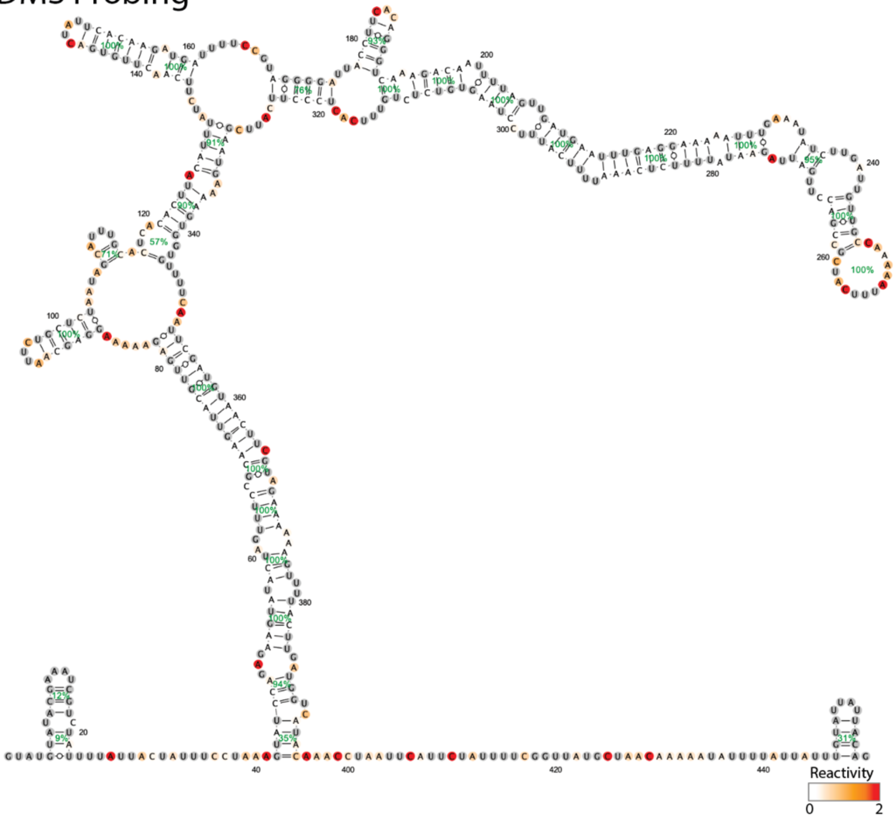

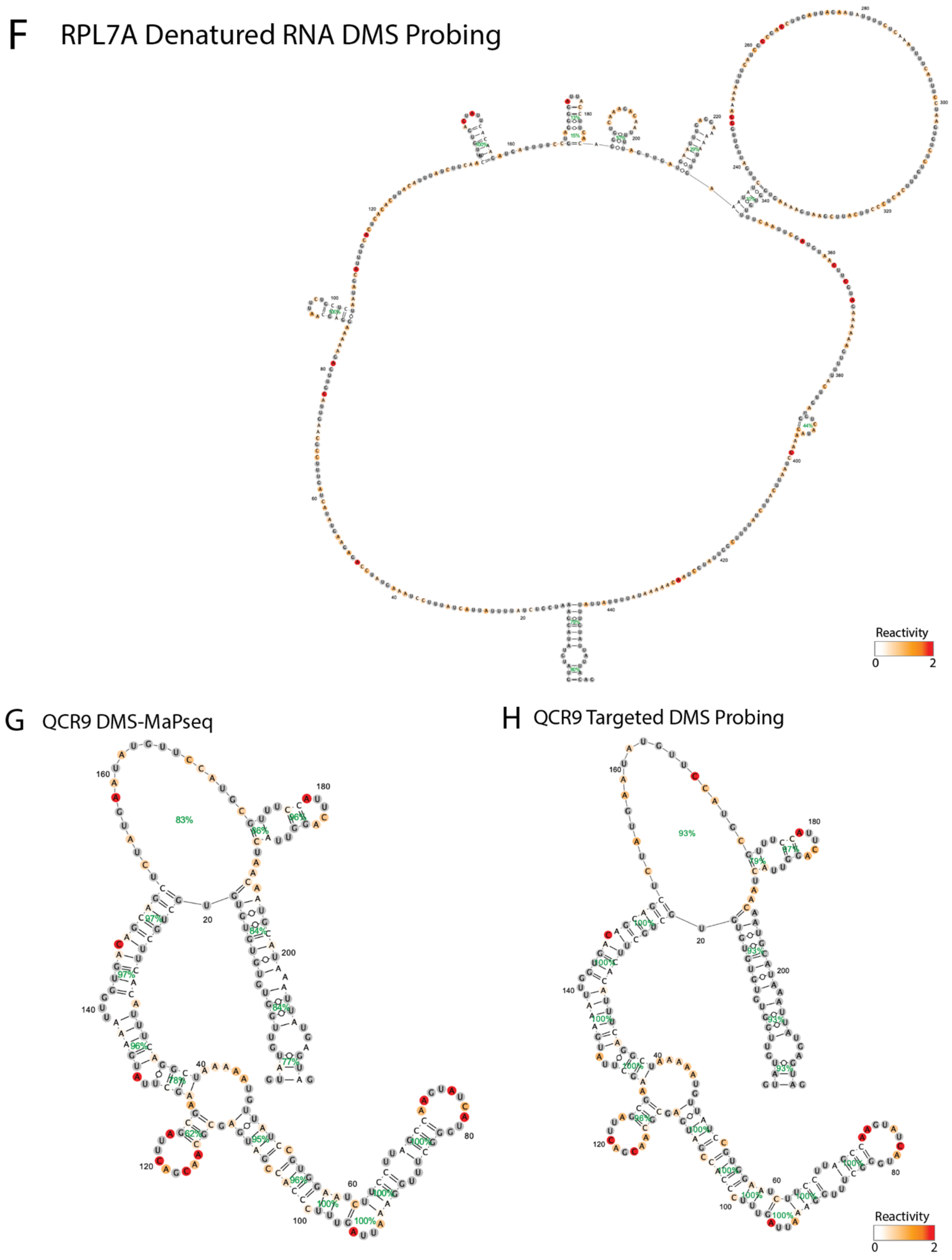

F RPL7A Denatured RNA DMS Probing
Reactivity
0
2
G QCR9 DMS-MaPseq
H QCR9 Targeted DMS Probing
Reactivity
0
2

I QCR9 Denatured RNA DMS Probing

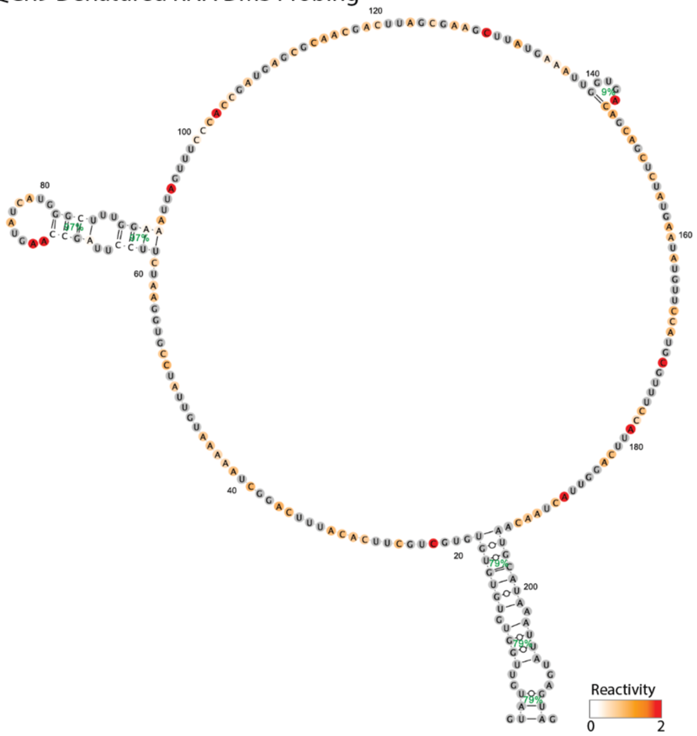

Supplementary Figure 2: Secondary structures predicted guided by DMS data from DMS-MaPseq, targeted DMS probing, and DMS probing of denatured RNA. **A)** *RPL28* intron DMS-MaPseq structure. **B)** *RPL28* intron targeted DMS probing structure. **C)** *RPL28* intron denatured RNA DMS probing structure. **D)** *RPL7A* intron DMS-MaPseq structure. **E)** *RPL7A* intron targeted DMS probing structure. **F)** *RPL7A* intron denatured RNA DMS probing structure. **G)** *QCR9* intron DMS-MaPseq structure. **H)** *QCR9* intron targeted DMS probing structure. **I)** *QCR9* intron denatured RNA DMS probing structure.

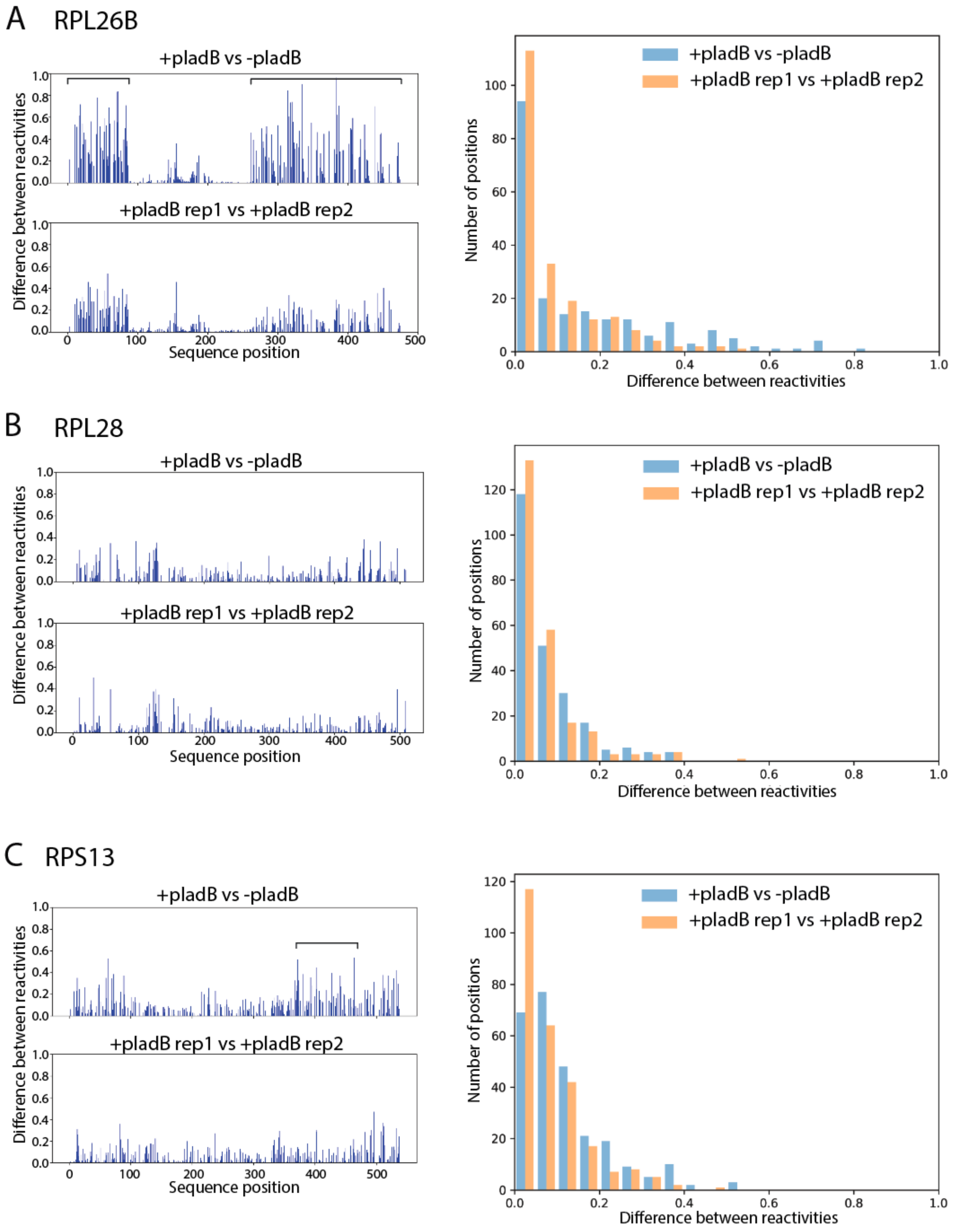

Supplementary Figure 3: Comparing reactivity values with and without pladB treatment for introns in **A)** *RPL26B*, **B)** *RPL28*, and **C)** *RPS13*. Left: the absolute value of the difference between reactivities (top row: comparing with and without pladB treatment; bottom row: comparing two replicates with pladB treatment.) Brackets highlight intervals including at least one position with > 0.5 absolute difference between reactivity values with vs without pladB treatment. Right: histograms summarizing the number of sequence positions with specified absolute differences between reactivity values.

RPL26B

A

-pladB +pladB

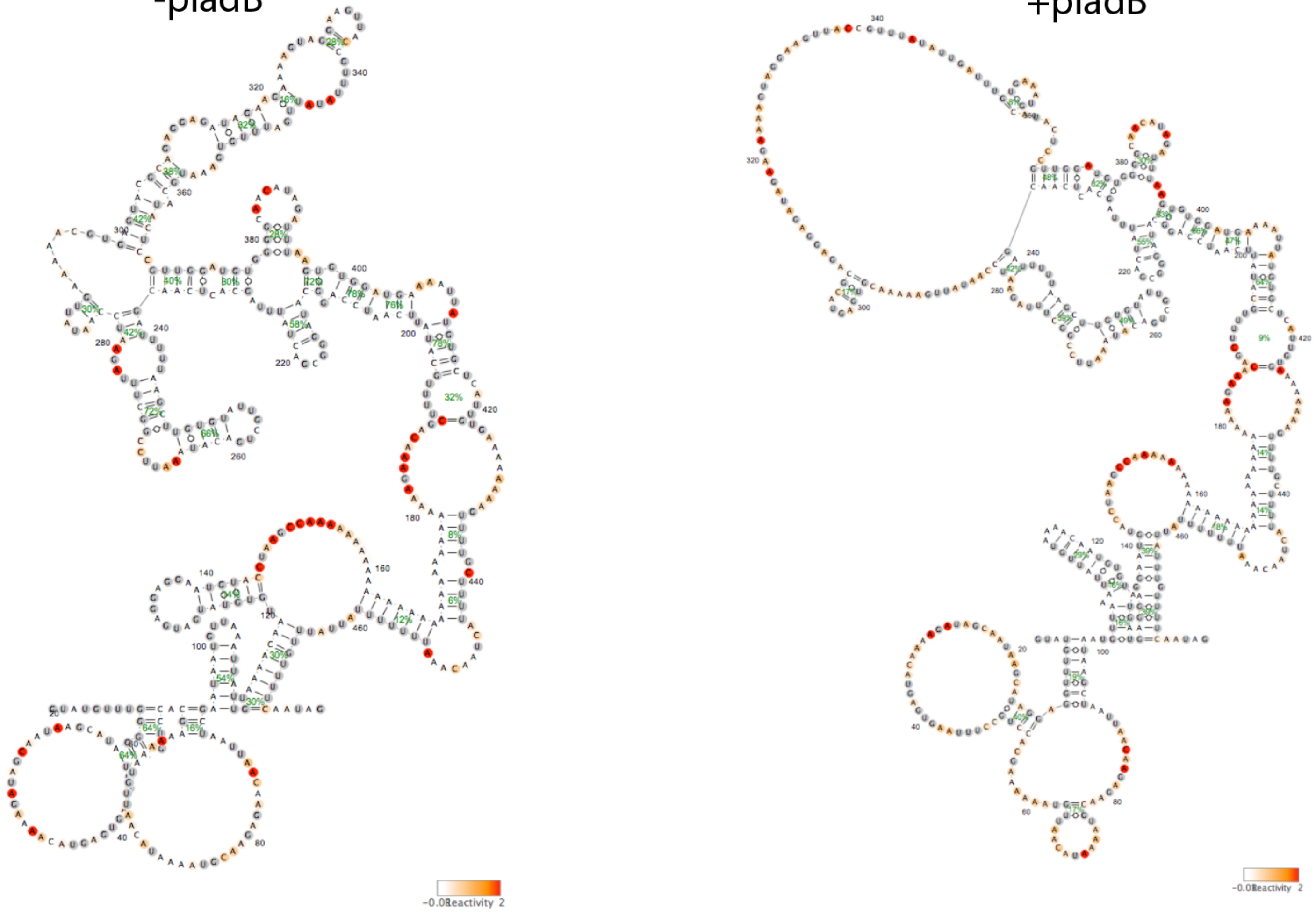

B

RPL28

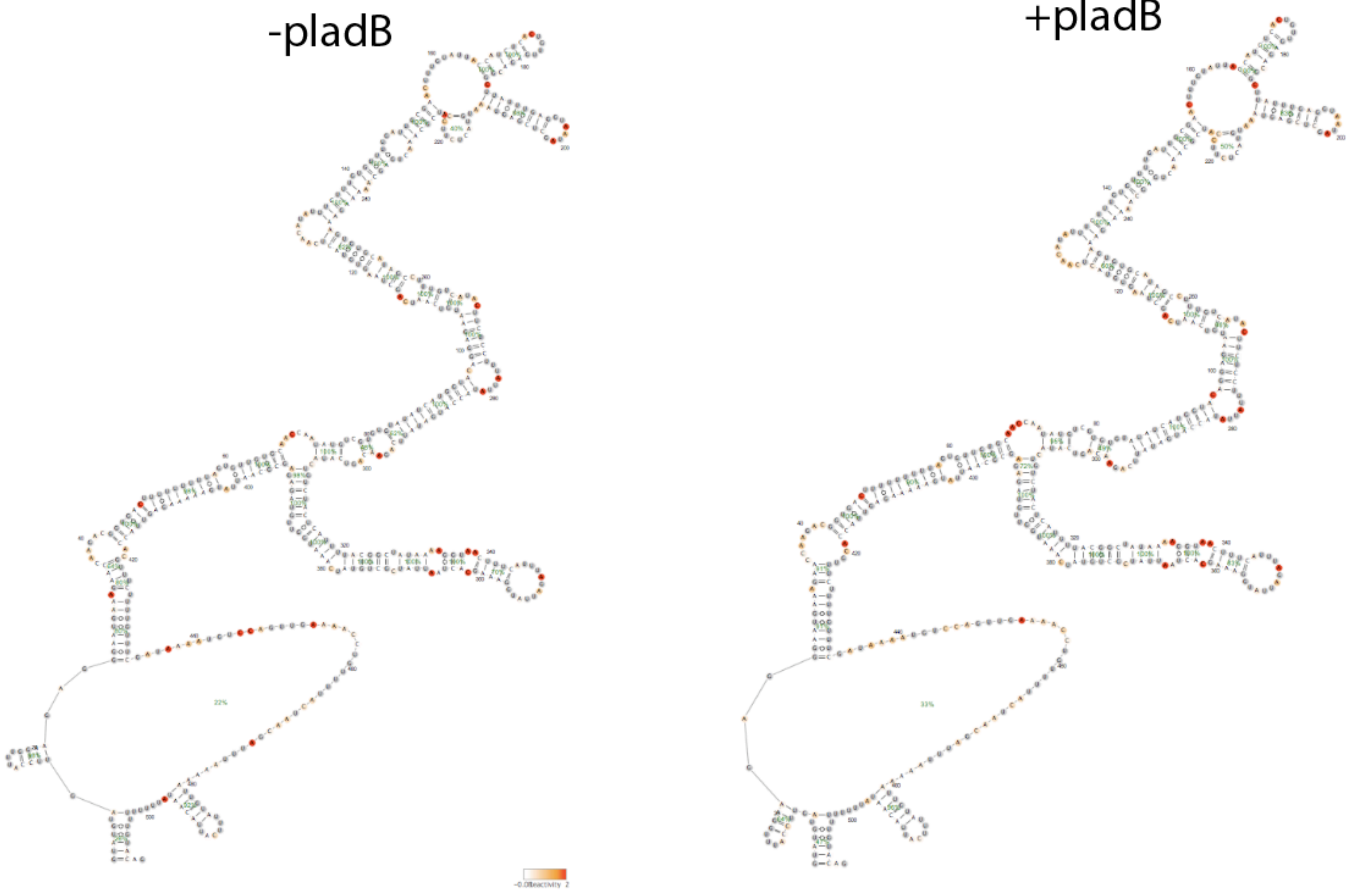

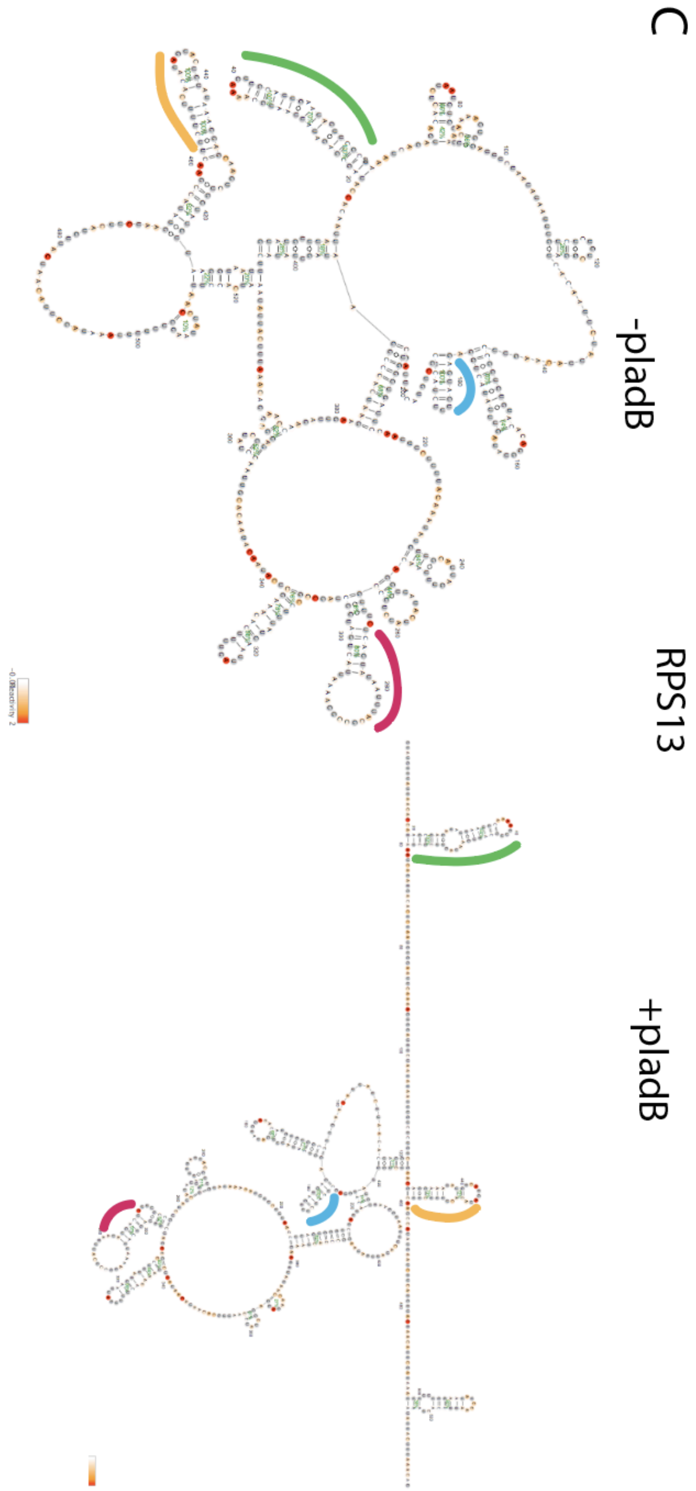

Supplementary Figure 4: Reactivity values, structure prediction, and bootstrapping probabilities with and without pladB treatment for introns in: **A)** *RPL26B*, **B)** *RPL28*, and **C)** *RPS13*. Common stems are highlighted in matching colors for *RPS13*.

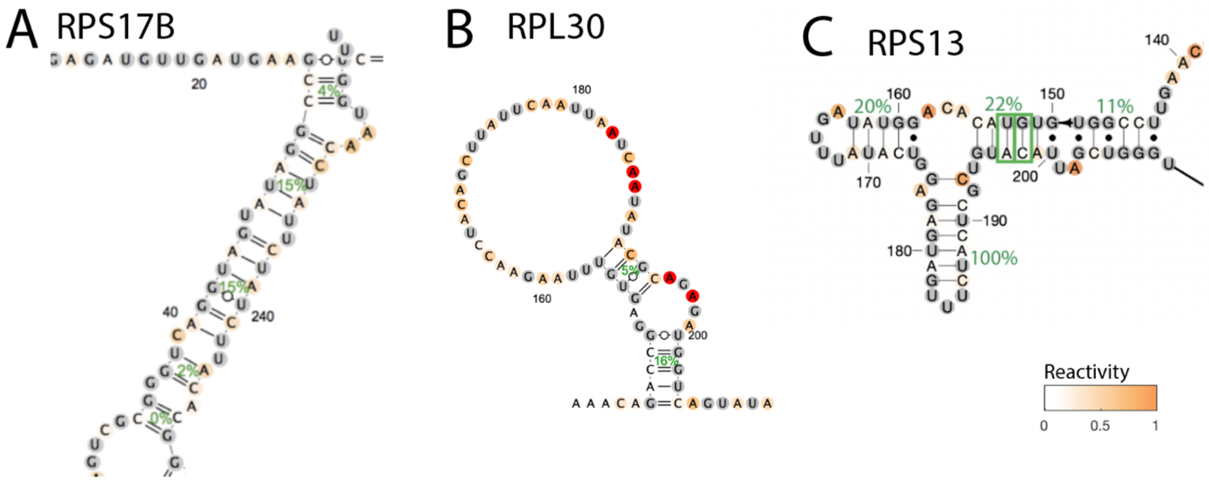

Supplementary Figure 5: Structures previously proposed from functional experiments or covariation scans with low DMS reactivity support. Secondary structures are colored by DMS reactivity and helix confidence estimates are depicted as green percentages. Covarying base pairs in the *RPS13* intron are indicated as green boxes.

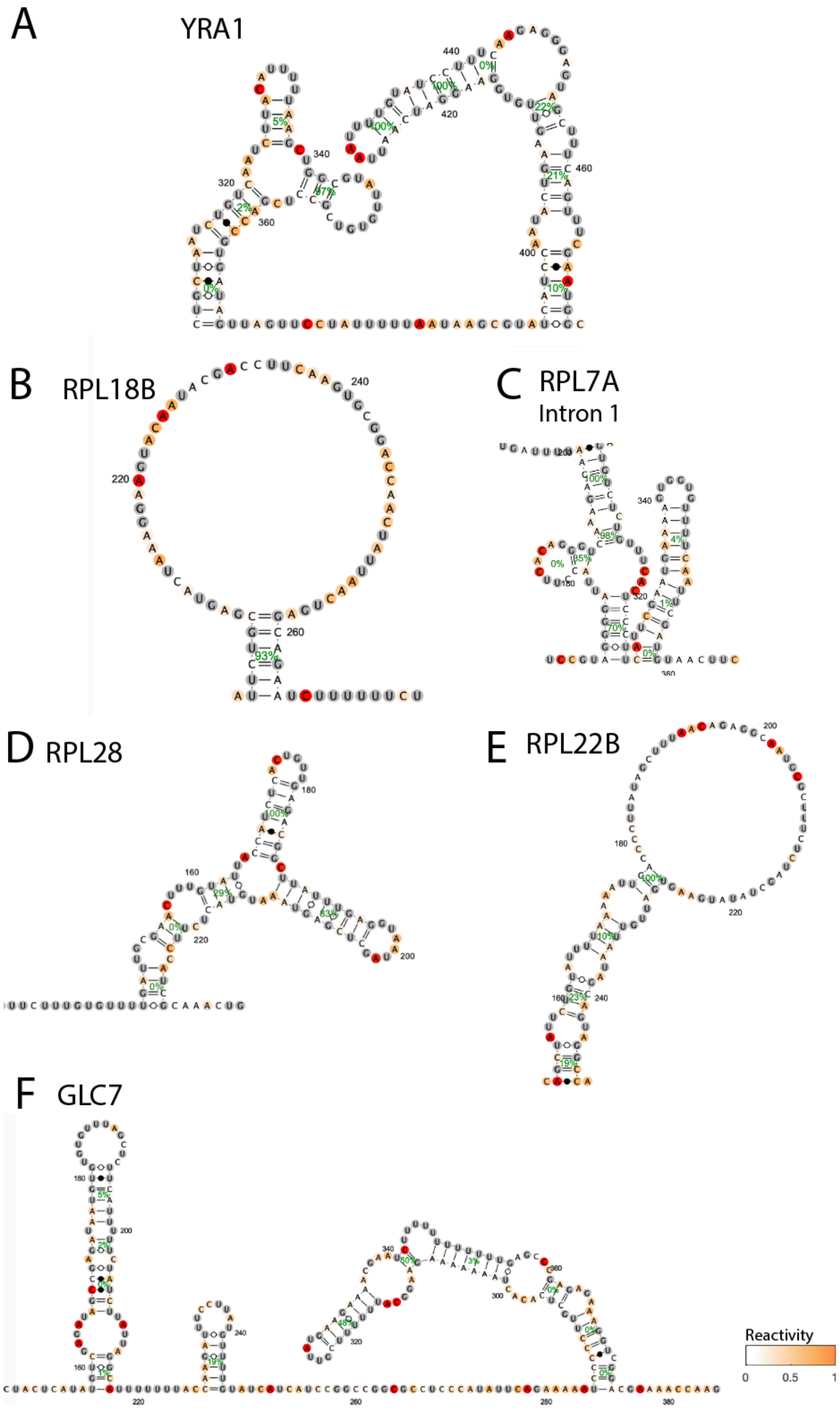

Supplementary Figure 6: DMS reactivity support for *in vivo* formation of structures from computational prediction based on evolutionary sequence alignments.

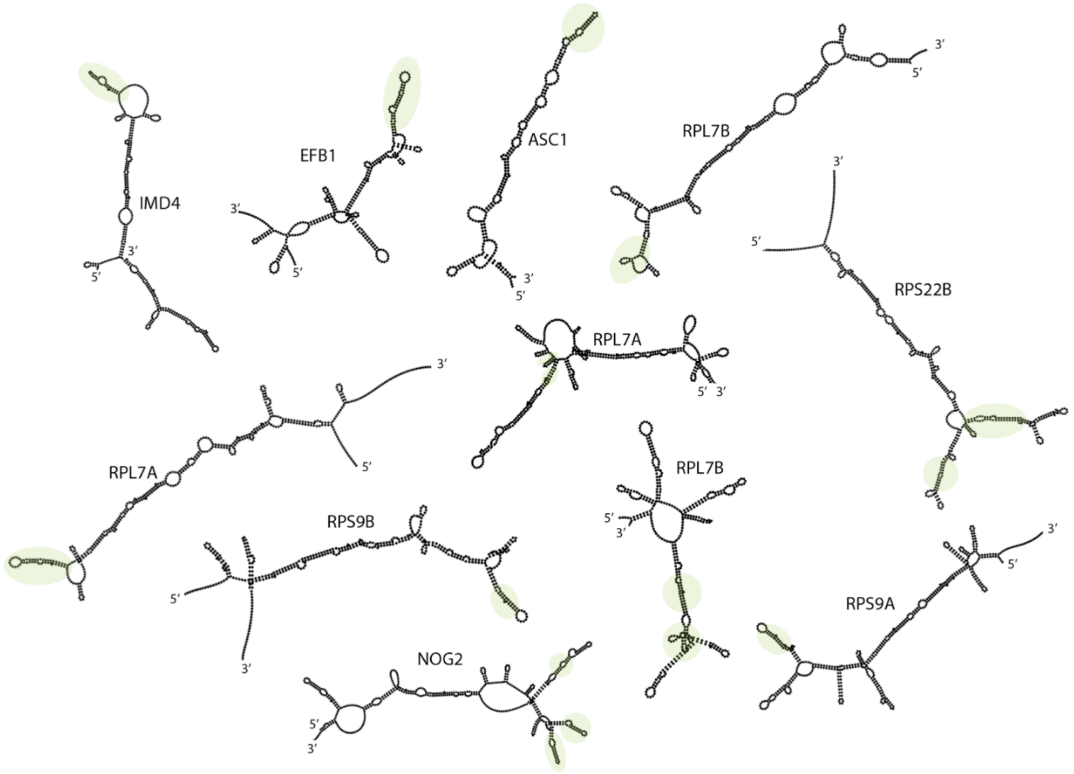

Supplementary Figure 7: Full gallery of introns longer than 200 nucleotides with at least one covarying base-pair in a stem of at least 3 base-pairs, only showing cases where the covariation data was consistent with the predicted minimum free energy structure.

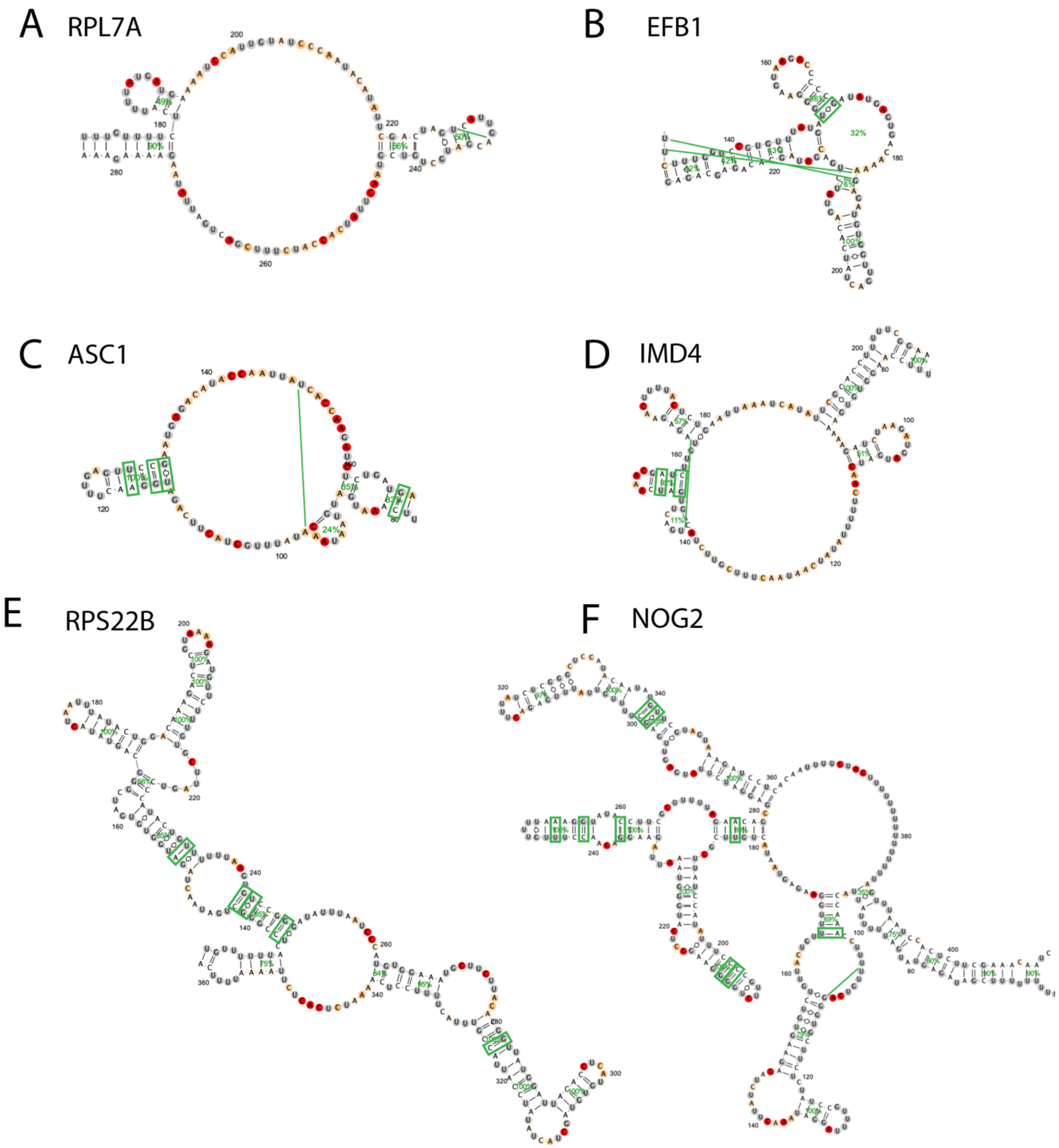

Supplementary Figure 8: DMS reactivity support for the *in vivo* formation of intron structures that include covariation in snoRNA regions. Covarying base-pairs are annotated in green boxes when they agree with base-pairs from the DMS-guided structure prediction, and lines when they include residues that are not base-paired in the DMS-guided structure prediction.

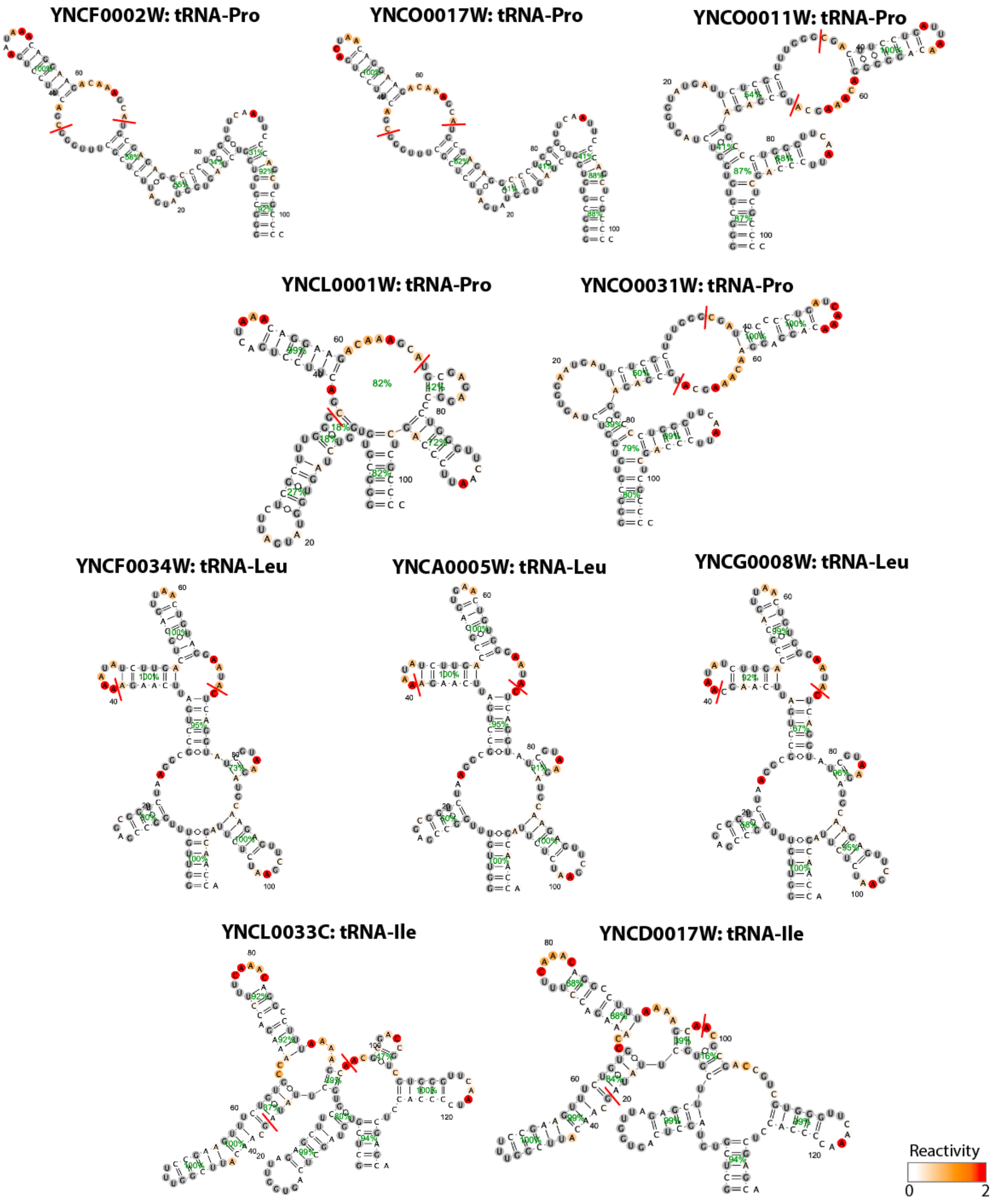

Supplementary Figure 9: DMS-guided structure predictions for tRNAs containing introns with length at least 30 nucleotides. Red lines indicate the excised introns' start and end positions.

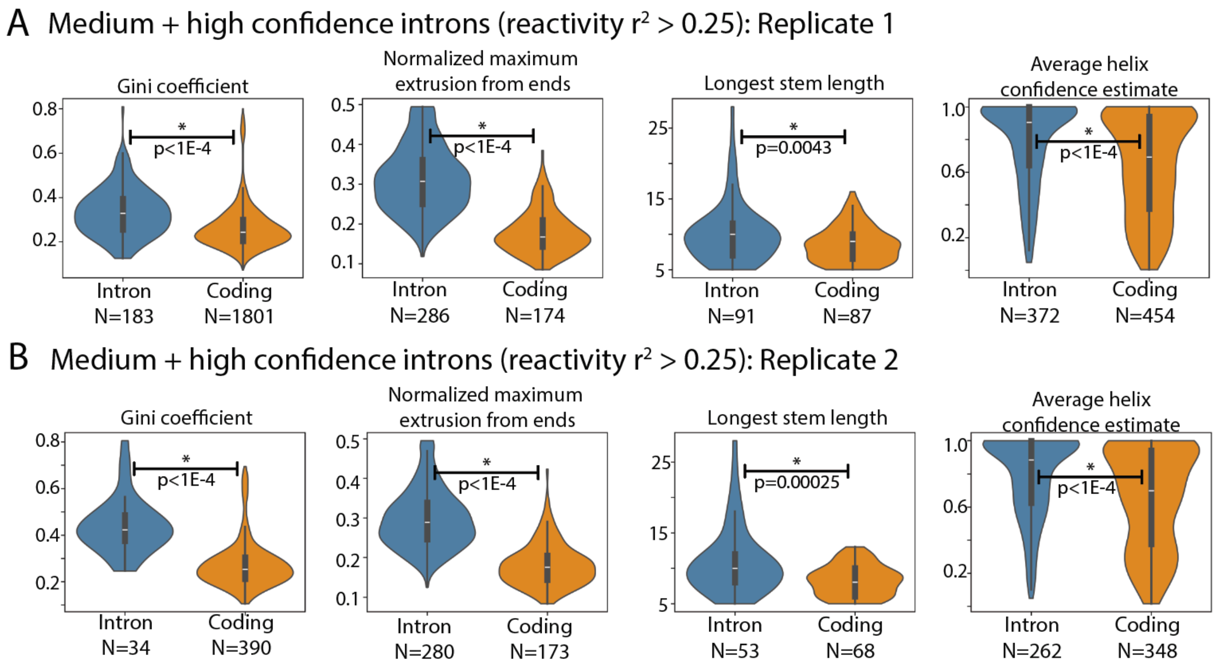

Supplementary Figure 10: Comparing intron and coding structural properties across replicate experiments. Comparisons are shown for **A)** medium and high confidence introns (between-replicate reactivity $r^2 > 0.25$) using data from the first DMS-MaPseq replicate, and **B)** medium and high confidence introns (between-replicate reactivity $r^2 > 0.25$) using data from the second DMS-MaPseq replicate. P-values for comparisons of secondary structure features between introns and coding regions were computed using one-sided Wilcoxon ranked sum tests. Box plots mark the median as the center white point and include a box from the 25th (Q1) to 75th (Q3) percentile, extending whiskers to the smallest and largest value that fall within 1.5 times the interquartile range below Q1 and above Q3.

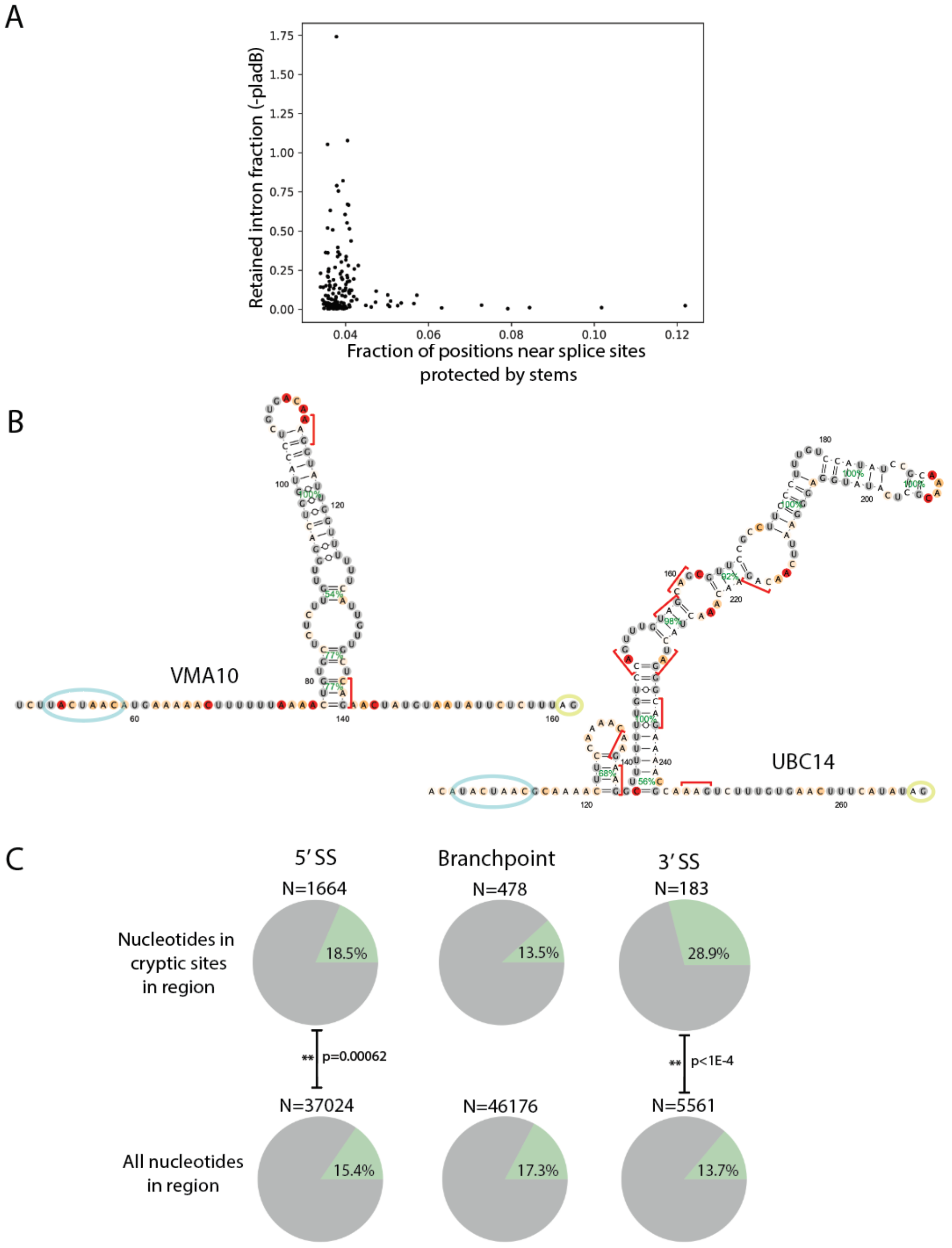

Supplementary Figure 11: Intron structures surrounding canonical and cryptic splice sites. **A)** The relationship between retained intron fraction and the fraction of positions surrounding the 5' splice site, branch point, and 3' splice site sequences that are occluded by high confidence stems. **B)** Example structures with downstream stems between the branch point (blue circle) and 3' splice site (yellow circle) that occlude cryptic 3' splice sites (red brackets). Secondary structures are colored by DMS reactivity and helix confidence estimates are depicted as green percentages. **C)** Comparison of the proportion of nucleotides protected by high confidence stems between nucleotides in cryptic splice sites versus nucleotides surrounding these sequences. Cryptic splice sites were identified by searching for sequences that matched other introns' splice site sequences in defined sequence intervals (details in Methods.) P-values are computed with Chi-squared tests on 2x2 contingency tables.

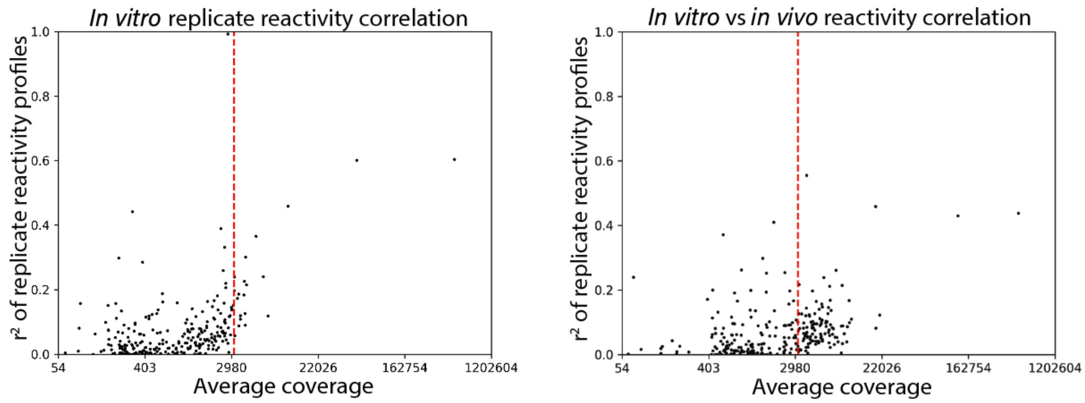

Supplementary Figure 12: Probing *in vitro* refolded RNA. Left: Pearson's correlation coefficient between *in vitro* DMS-MaPseq replicates for each intron in *S. cerevisiae* versus the average sequencing coverage between replicates. Right: correlation between reactivity values for introns probed in *in vivo* and *in vitro* DMS-MaPseq, versus the average coverage in these experiments. The vertical red line indicates the coverage cutoff used for analysis of *in vivo* samples.

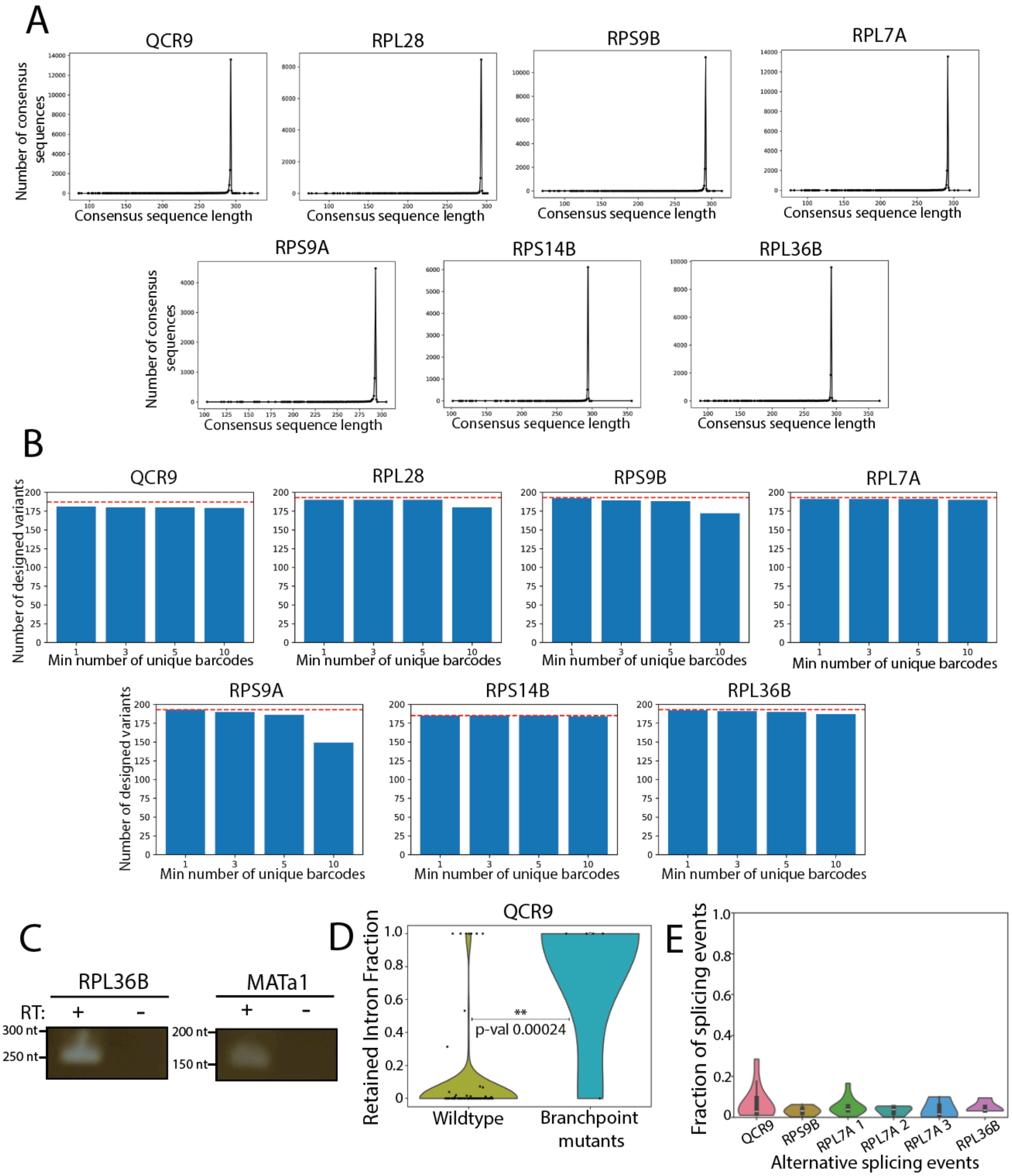

Supplementary Figure 13: Structure variant library gDNA and RNA sequencing validation. **A)** Histogram of consensus sequence lengths determined for each barcode from gDNA sequencing. **B)** Number of designed variants assigned to at least 1, 3, 5, or 10 unique barcodes from gDNA sequencing. Red dashed line indicates total number of designed variants per construct. **C)** RT-PCR for control regions (RNA intervals in *RPL36B* and *MATa1*) demonstrating depletion of gDNA from targeted RNA-sequencing library preparation. **D)** Accumulation of retained introns for *QCR9* variants with branch point mutations. P-value from a two-sided permutation test for

the mean statistic, comparing 143 wildtype samples with 5 branchpoint mutants. **E)** For each of six recurring alternative splicing events across 4 introns, we identified the variant sequences for which these events were observed. These violin plots depict the fraction of splicing events that were alternatively spliced for transcripts from these variant sequences. Box plots mark the median as the center white point and include a box from the 25th (Q1) to 75th (Q3) percentile, extending whiskers to the smallest and largest value that fall within 1.5 times the interquartile range below Q1 and above Q3.

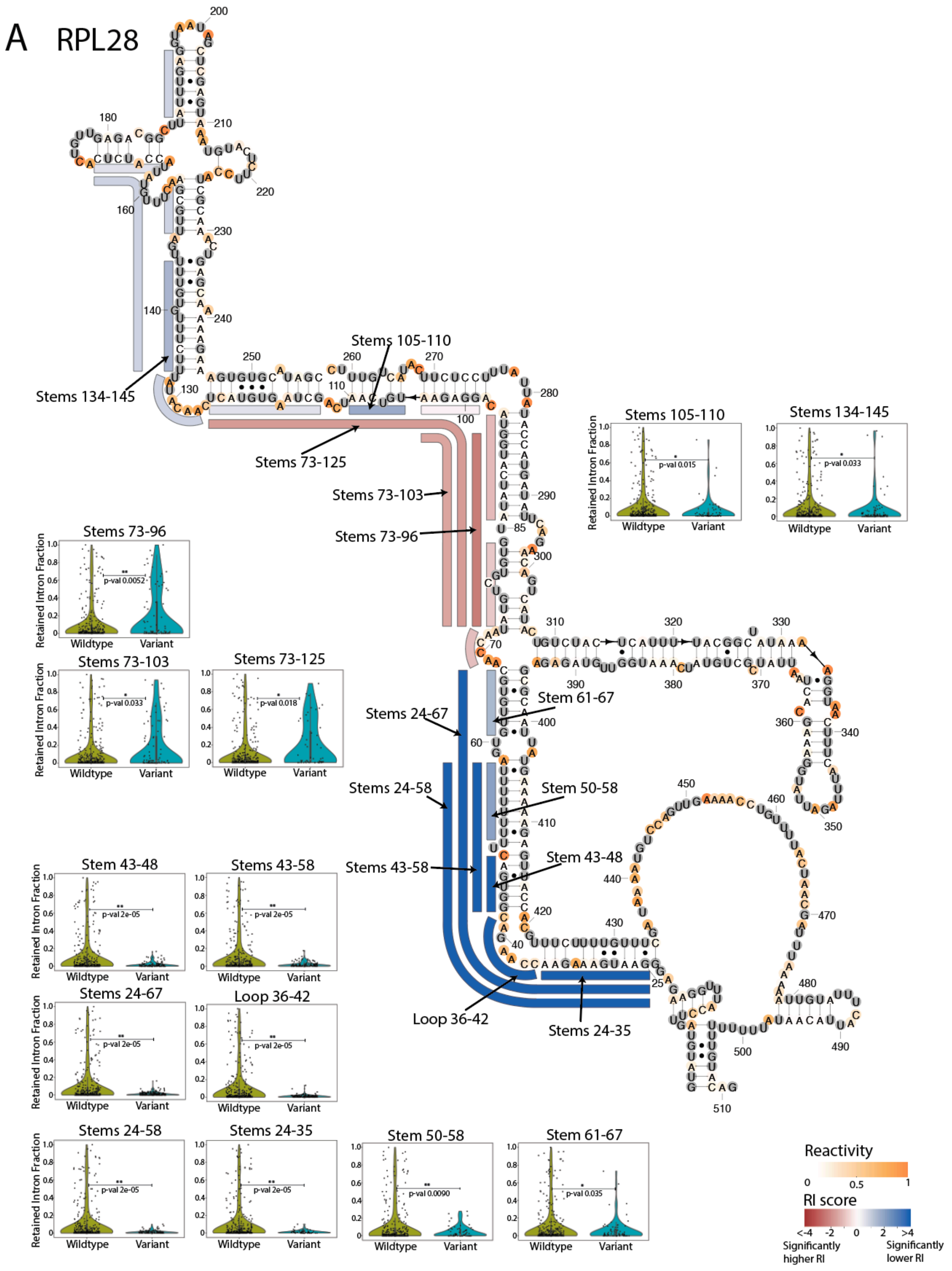
A
RPL28
Stems 134-145
Stems 105-110
Stems 73-125
Stems 73-103
Stems 73-96
Stems 24-67
Stem 61-67
Stems 24-58
Stem 50-58
Stems 43-58
Stem 43-48
Loop 36-42
Stems 24-35
Retained Intron Fraction
Wildtype
Variant
p-val 0.015
p-val 0.033
p-val 0.0052
p-val 0.018
p-val 2e-05
p-val 0.0090
p-val 0.035
Reactivity
RI score
Significantly higher RI
Significantly lower RI

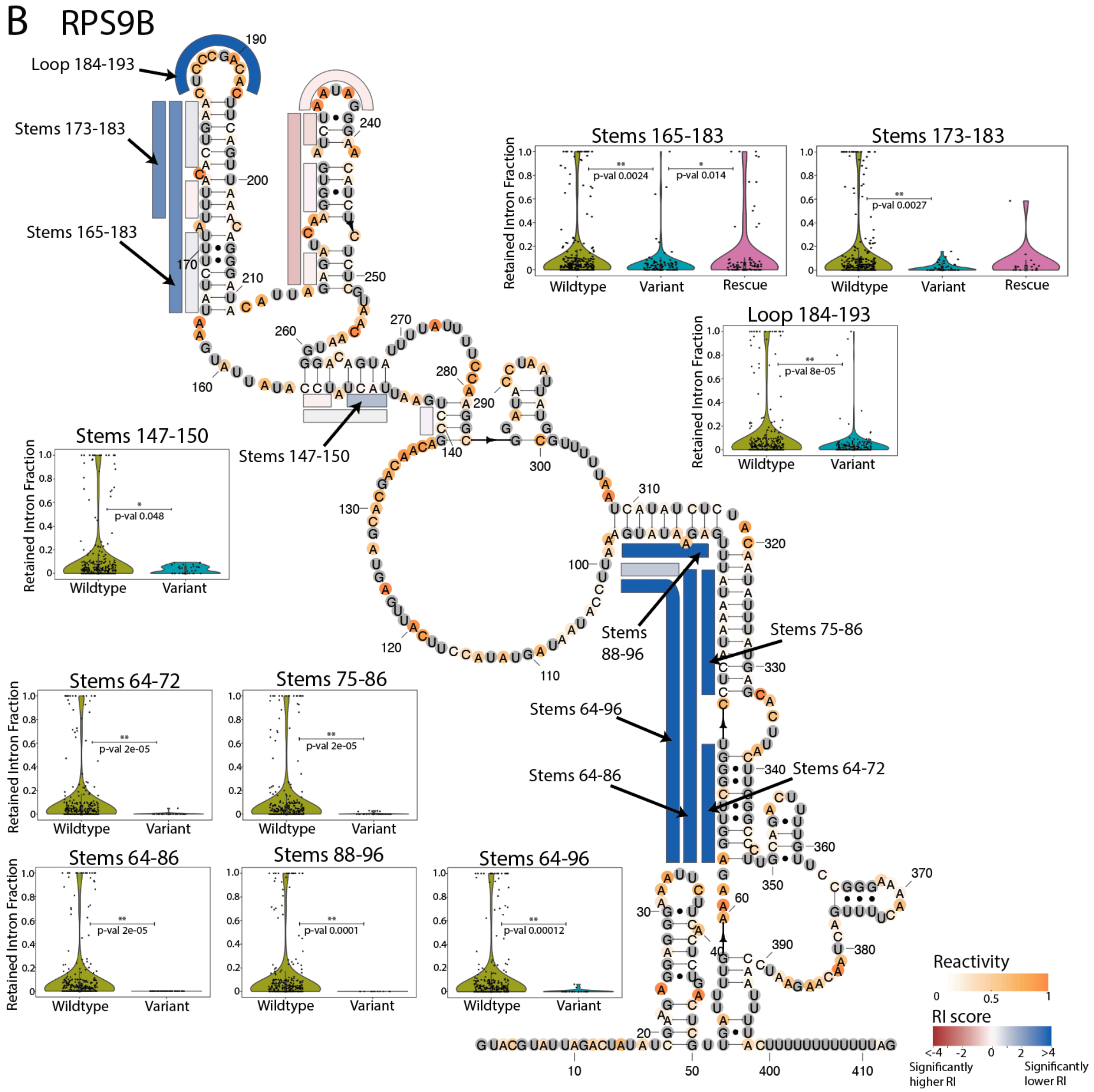

B
RPS9B
Loop 184-193
Stems 173-183
Stems 165-183
Stems 147-150
Stems 88-96
Stems 75-86
Stems 64-96
Stems 64-86
Stems 64-72
Stems 165-183
p-val 0.0024
p-val 0.014
Stems 173-183
p-val 0.0027
Loop 184-193
p-val 8e-05
Stems 147-150
p-val 0.048
Stems 64-72
p-val 2e-05
Stems 75-86
p-val 2e-05
Stems 64-86
p-val 2e-05
Stems 88-96
p-val 0.0001
Stems 64-96
p-val 0.00012
Retained Intron Fraction
Wildtype
Variant
Rescue
Reactivity
RI score
Significantly higher RI
Significantly lower RI

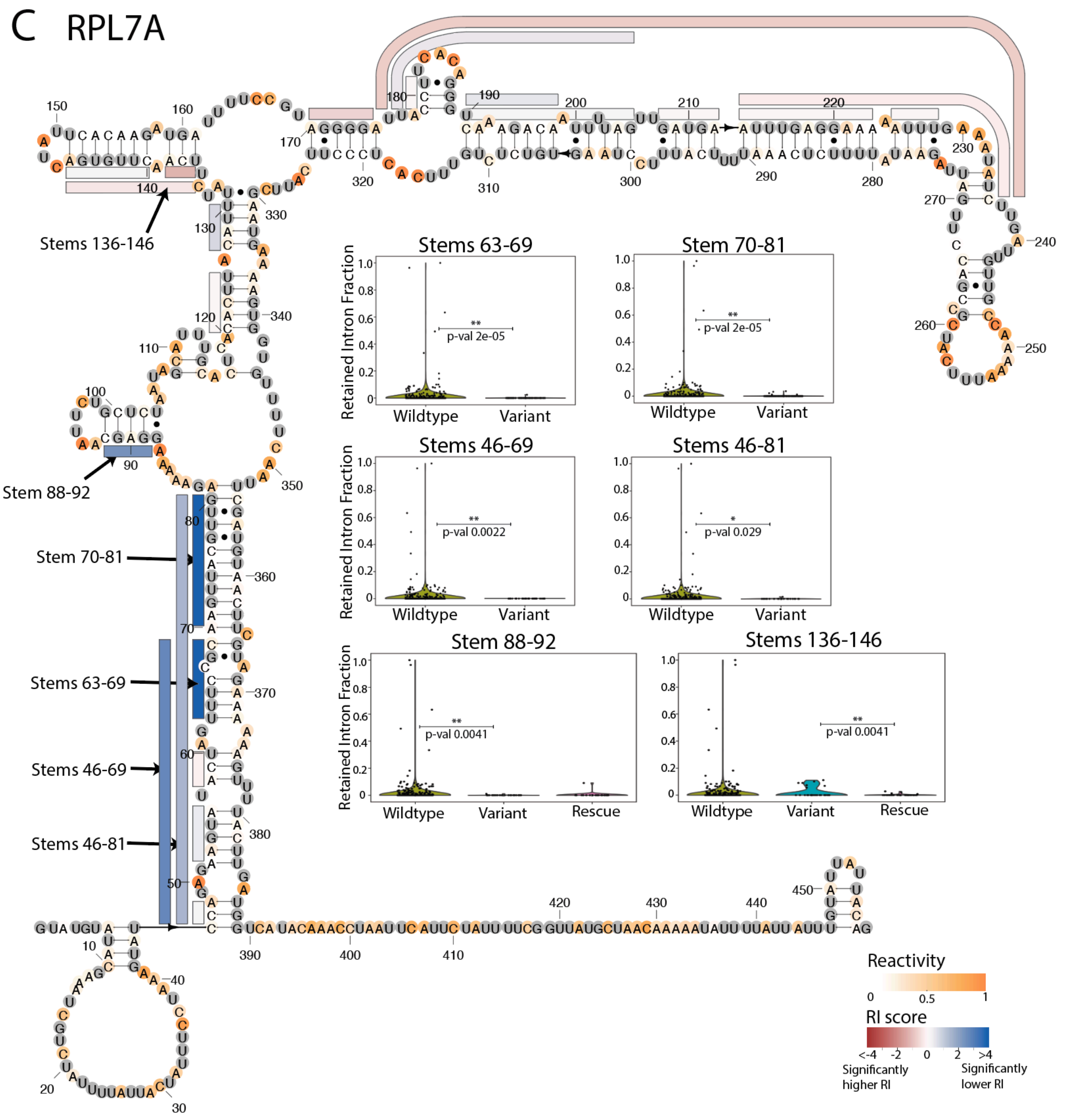
C RPL7A
Stems 136-146
Stem 88-92
Stem 70-81
Stems 63-69
Stems 46-69
Stems 46-81
Stems 63-69
Stem 70-81
Stems 46-69
Stems 46-81
Stem 88-92
Stems 136-146
Retained Intron Fraction
Wildtype
Variant
Rescue
p-val 2e-05
p-val 2e-05
p-val 0.0022
p-val 0.029
p-val 0.0041
p-val 0.0041
Reactivity
RI score
Significantly higher RI
Significantly lower RI

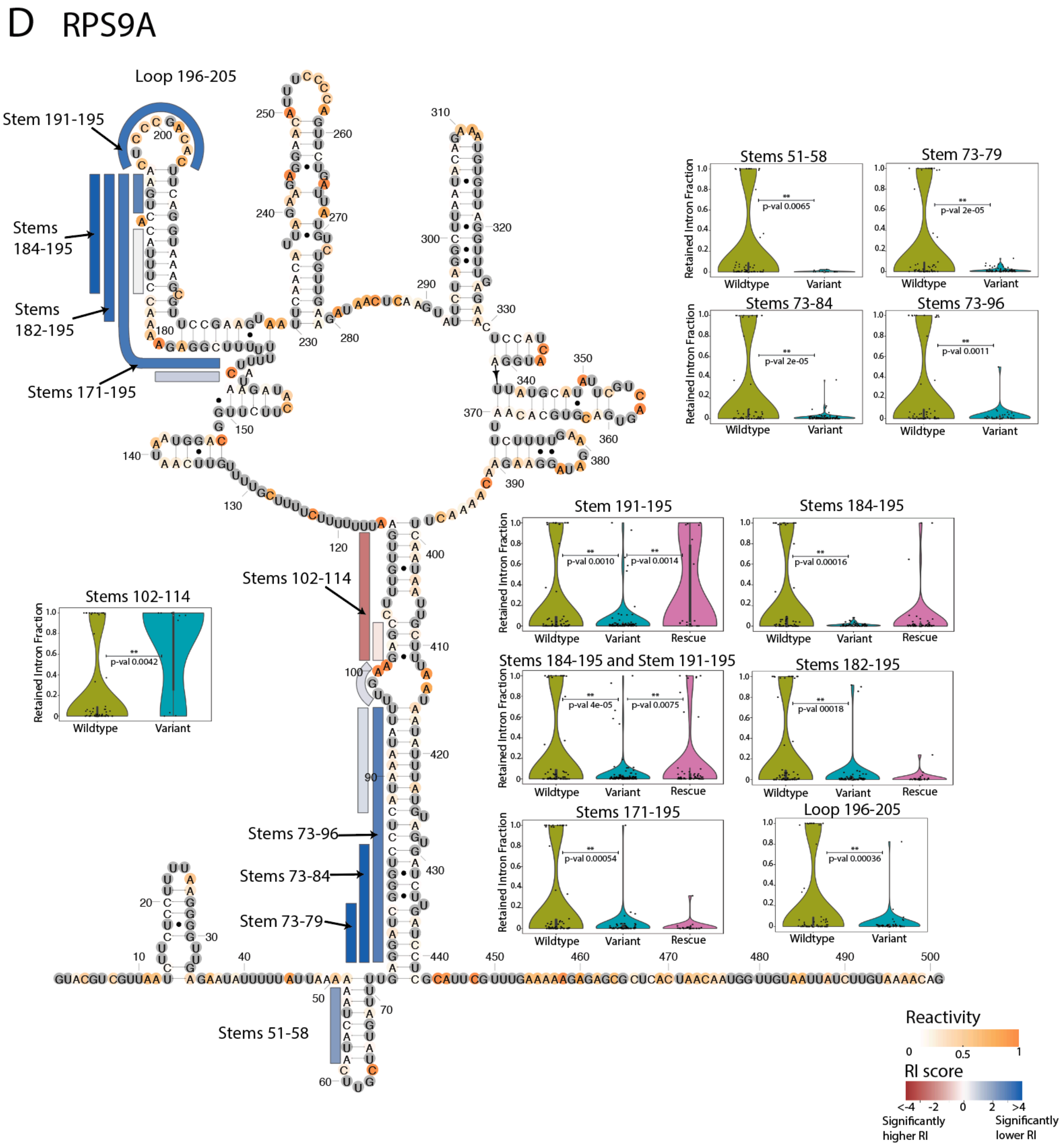
D RPS9A
Loop 196-205
Stem 191-195
Stems 184-195
Stems 182-195
Stems 171-195
Stems 102-114
Stems 73-96
Stems 73-84
Stem 73-79
Stems 51-58
Stems 51-58
Stem 73-79
Stems 73-84
Stems 73-96
Stem 191-195
Stems 184-195
Stems 184-195 and Stem 191-195
Stems 182-195
Stems 171-195
Loop 196-205
Stems 102-114
Retained Intron Fraction
Wildtype
Variant
Rescue
Reactivity
RI score
Significantly higher RI
Significantly lower RI

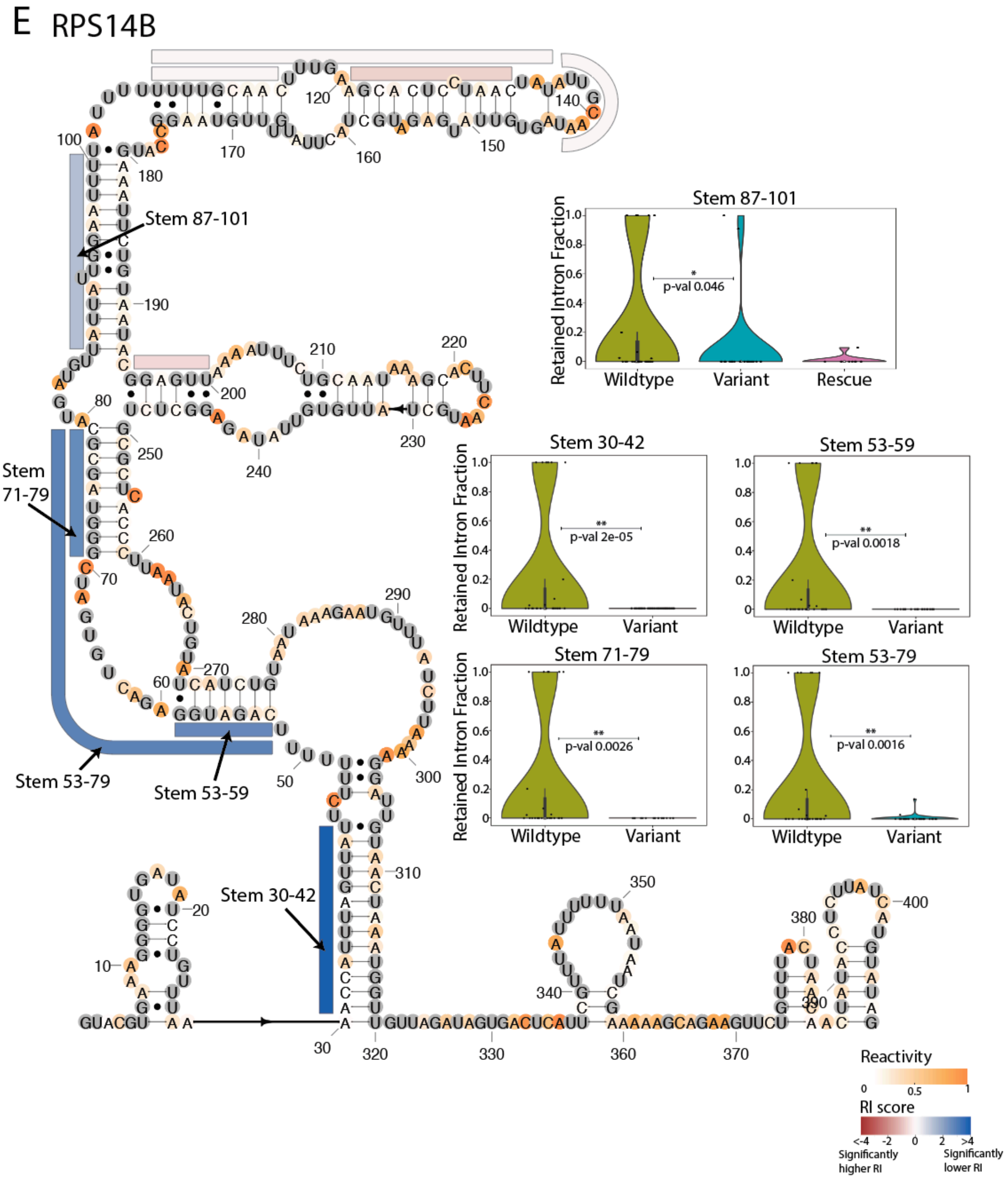
E RPS14B
Stem 87-101
Stem 71-79
Stem 53-79
Stem 53-59
Stem 30-42
Stem 87-101
p-val 0.046
Retained Intron Fraction
Wildtype
Variant
Rescue
Stem 30-42
p-val 2e-05
Stem 53-59
p-val 0.0018
Stem 71-79
p-val 0.0026
Stem 53-79
p-val 0.0016
Reactivity
RI score
Significantly higher RI
Significantly lower RI

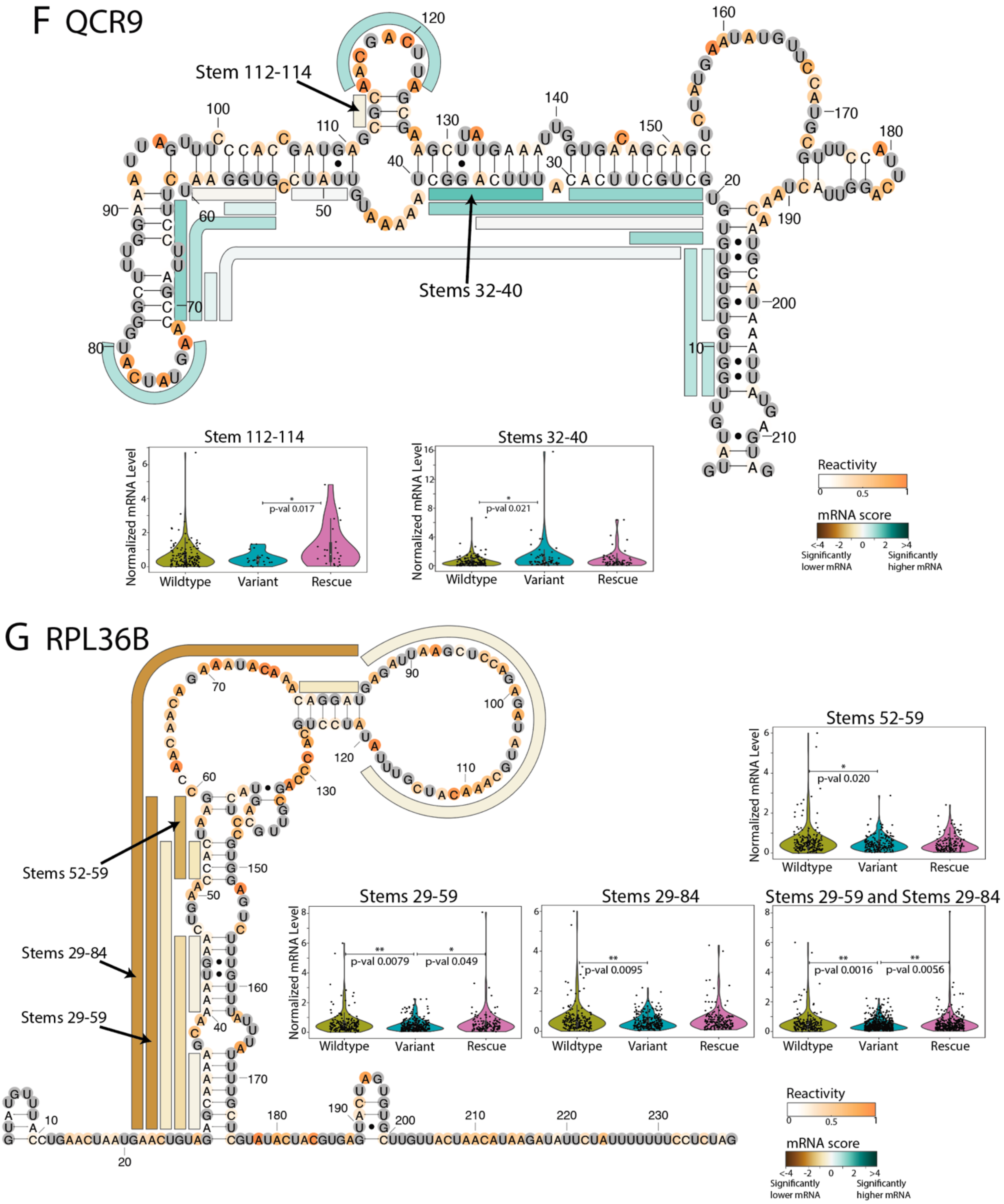

Supplementary Figure 14: Effects of structure variants on retained intron levels for **A)** *RPL28*, **B)** *RPS9B*, **C)** *RPL7A*, **D)** *RPS9A*, **E)** *RPS14B*, and effects of structure variants on normalized mRNA levels in **F)** *QCR9*, and **G)** *RPL36B*. For a given stem set or loop, violin plots depict data for the wildtype sequence and all variant sequences, with points for each unique barcode. Data for rescue sequences are shown when included in the intron library. p-values (computed by two-

sided permutation tests) are indicated for comparisons between wildtype and variant sequences, and between variant and rescue sequences. The number of datapoints for each variant sequence along with source data for plots are included in Supplementary Table 4. Box plots mark the median as the center white point and include a box from the 25th (Q1) to 75th (Q3) percentile, extending whiskers to the smallest and largest value that fall within 1.5 times the interquartile range below Q1 and above Q3. Secondary structures are colored by reactivity data, and bars alongside the secondary structure indicate stem and loop disruption sets, with each bar representing variant sequences mutating nucleotides across the full extent of the bar. These bars are colored by the retained intron (RI) score (A-E) and mRNA scores (F-G) for the interval. The RI score is the negative log(p-value) comparing RI values between wildtype and variant sequences, and the sign indicates the effect direction, with positive values (shown as blue) for lower variant RI compared to wildtype, and negative values (shown as red) for higher variant RI. Similarly, the mRNA score is computed as the negative log(p-value) comparing normalized mRNA levels between wildtype and variant sequences, with positive (green) values indicating higher variant mRNA levels, and negative (brown) values indicating lower variant mRNA levels.

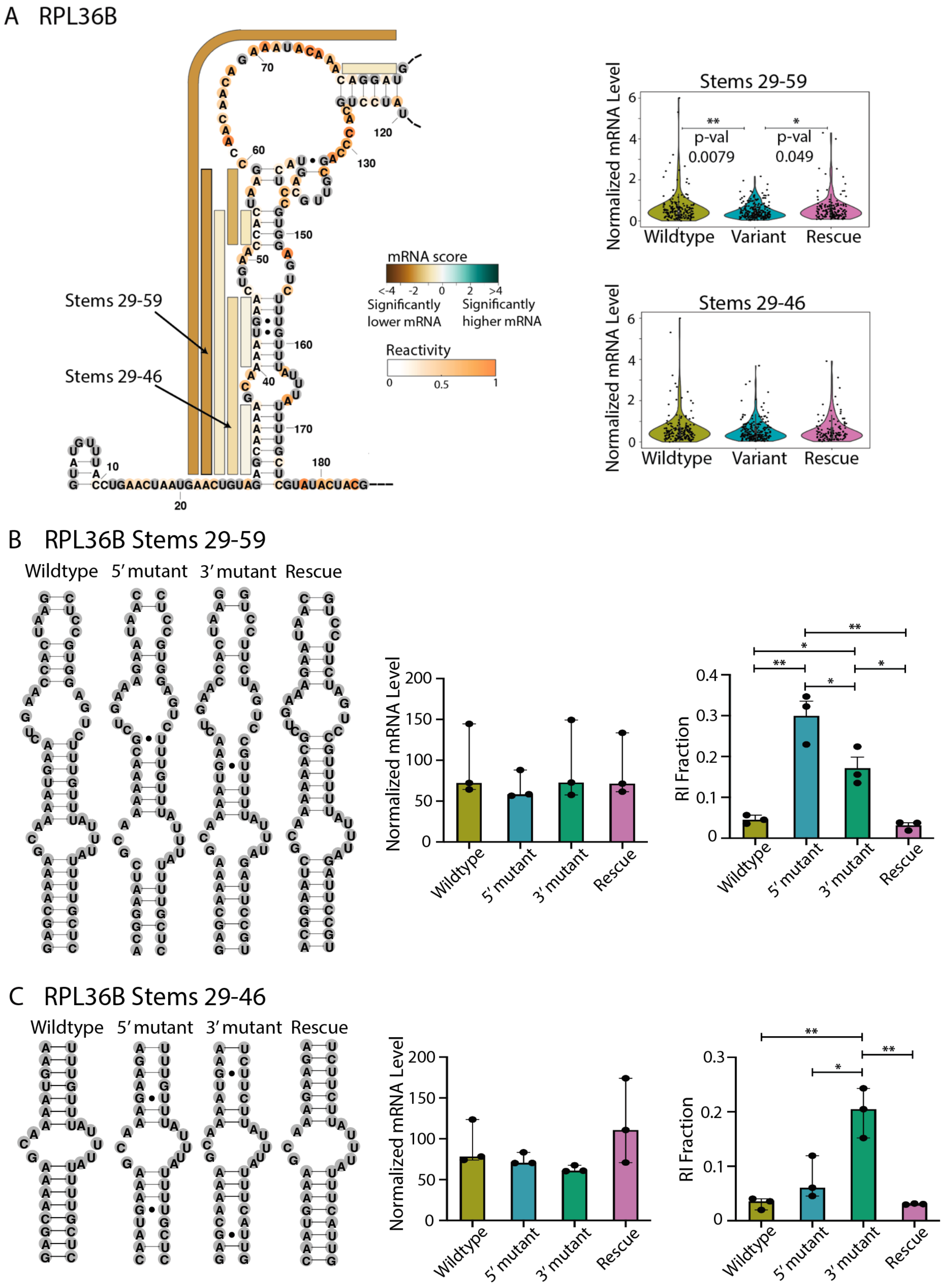
A RPL36B
Stems 29-59
Stems 29-46
mRNA score
<-4 -2 0 2 >4
Significantly lower mRNA
Significantly higher mRNA
Reactivity
0 0.5 1
Normalized mRNA Level
Stems 29-59
p-val 0.0079
p-val 0.049
Wildtype Variant Rescue
Stems 29-46
B RPL36B Stems 29-59
Wildtype 5′ mutant 3′ mutant Rescue
Normalized mRNA Level
RI Fraction
C RPL36B Stems 29-46
Wildtype 5′ mutant 3′ mutant Rescue
Normalized mRNA Level
RI Fraction

Supplementary Figure 15: Assessing the effects of stem variant and rescue sequences on stem sets in the *RPL36B* intron with VARS-seq and RT-qPCR. **A)** The effects of structure variants on normalized mRNA levels for two regions of the *RPL36B* intron. For a given stem set or loop, violin plots depict data for the wildtype sequence and all variant sequences, with black points for each unique barcode. p-values are indicated for comparisons between wildtype and variant sequence sets, and between variant and rescue sequence sets. For stems 29-59, comparisons involve 180 wildtype sequences, 282 variant sequences, and 128 rescue sequences. For stems 29-46, comparisons involve 180 wildtype sequences, 224 variant sequences, and 119 rescue sequences. p-values are computed using two-sided permutation tests for the difference in mean statistic. Box plots mark the median as the center white point and include a box from the 25th (Q1) to 75th (Q3) percentile, extending whiskers to the smallest and largest value that fall within 1.5 times the interquartile range below Q1 and above Q3. Secondary structures are colored by reactivity data. Bars alongside the secondary structure indicate stem and loop disruption sets, with each bar representing a set of variant sequences mutating nucleotides across the full extent of the bar. These bars are colored by the mRNA score for the corresponding stem or loop disruption set. The mRNA score is computed as the negative log(p-value) when comparing normalized mRNA levels between wildtype and variant sequences, with positive (green) values indicating higher variant mRNA levels, and negative (brown) values indicating lower variant mRNA levels. **B, C)** RI fractions as measured by RT-qPCR for individual strains representing two sets of wildtype, variant, and rescue sequences for *RPL36B* stem sets. Stem variants are shown on the left, and RT-qPCR data are shown for 3 biological replicates on the right. Data are presented as median values with a 95% confidence interval marked. p-values are computed with 2-way ANOVA tests with multiple comparisons, with *p-value < 0.05, and **p-value < 0.001. Exact p-values for the RI fractions in B) are as follows: p=0.0003 for wildtype vs 5' mutant, p=0.011 for wildtype vs 3' mutant, p=0.0002 for 5' mutant vs rescue, p=0.011 for 5' mutant vs 3' mutant, and p=0.0068 for 3' mutant vs rescue. Exact p-values for the RI fractions in C) are as follows: p=0.0004 for wildtype vs 3' mutant, p=0.0022 for 5' mutant vs 3' mutant, and p=0.0004 for 3' mutant vs rescue.

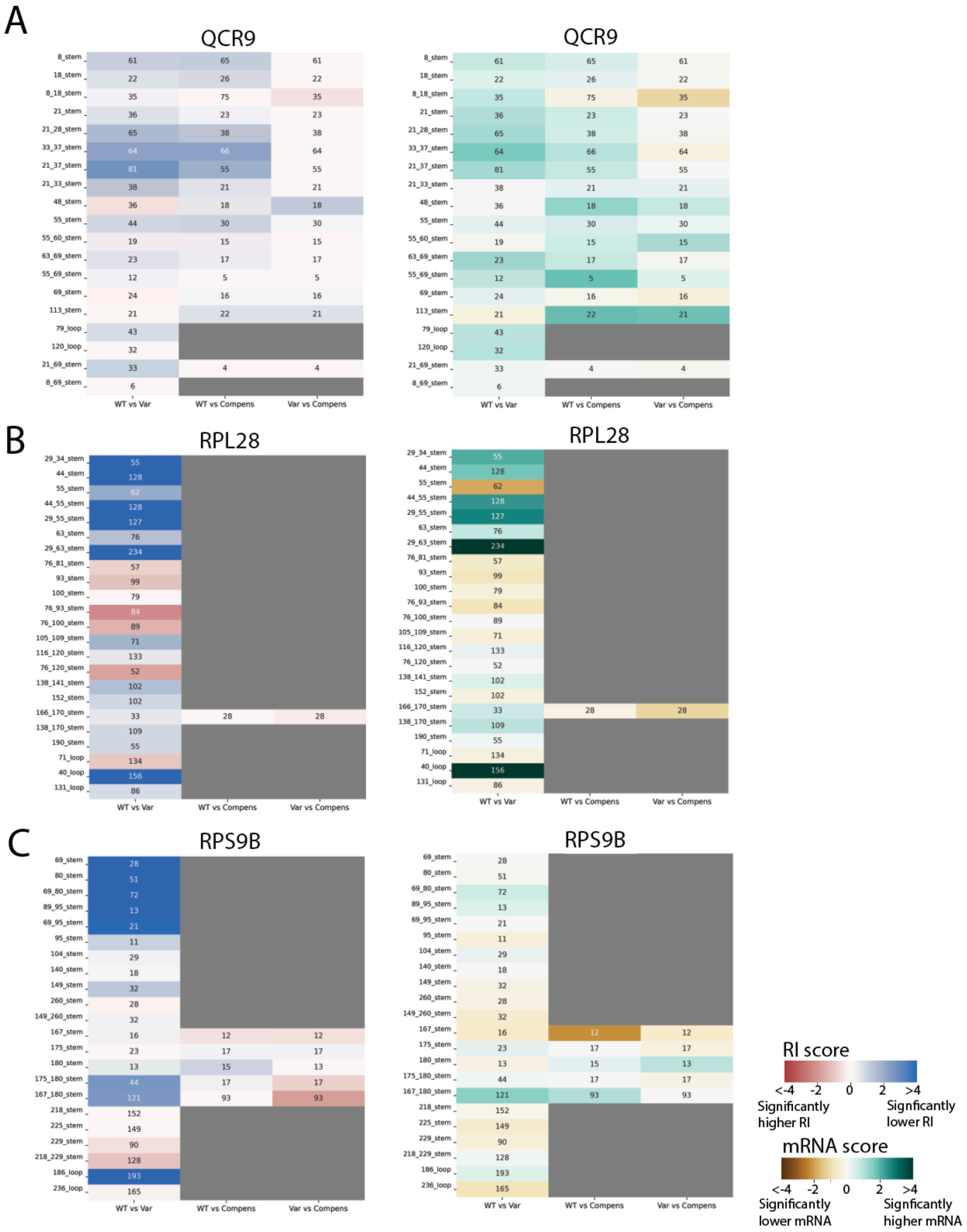
A
QCR9
QCR9
B
RPL28
RPL28
C
RPS9B
RPS9B
WT vs Var
WT vs Compens
Var vs Compens
RI score
<-4 -2 0 2 >4
Significantly higher RI
Signficantly lower RI
mRNA score
<-4 -2 0 2 >4
Significantly lower mRNA
Signficantly higher mRNA

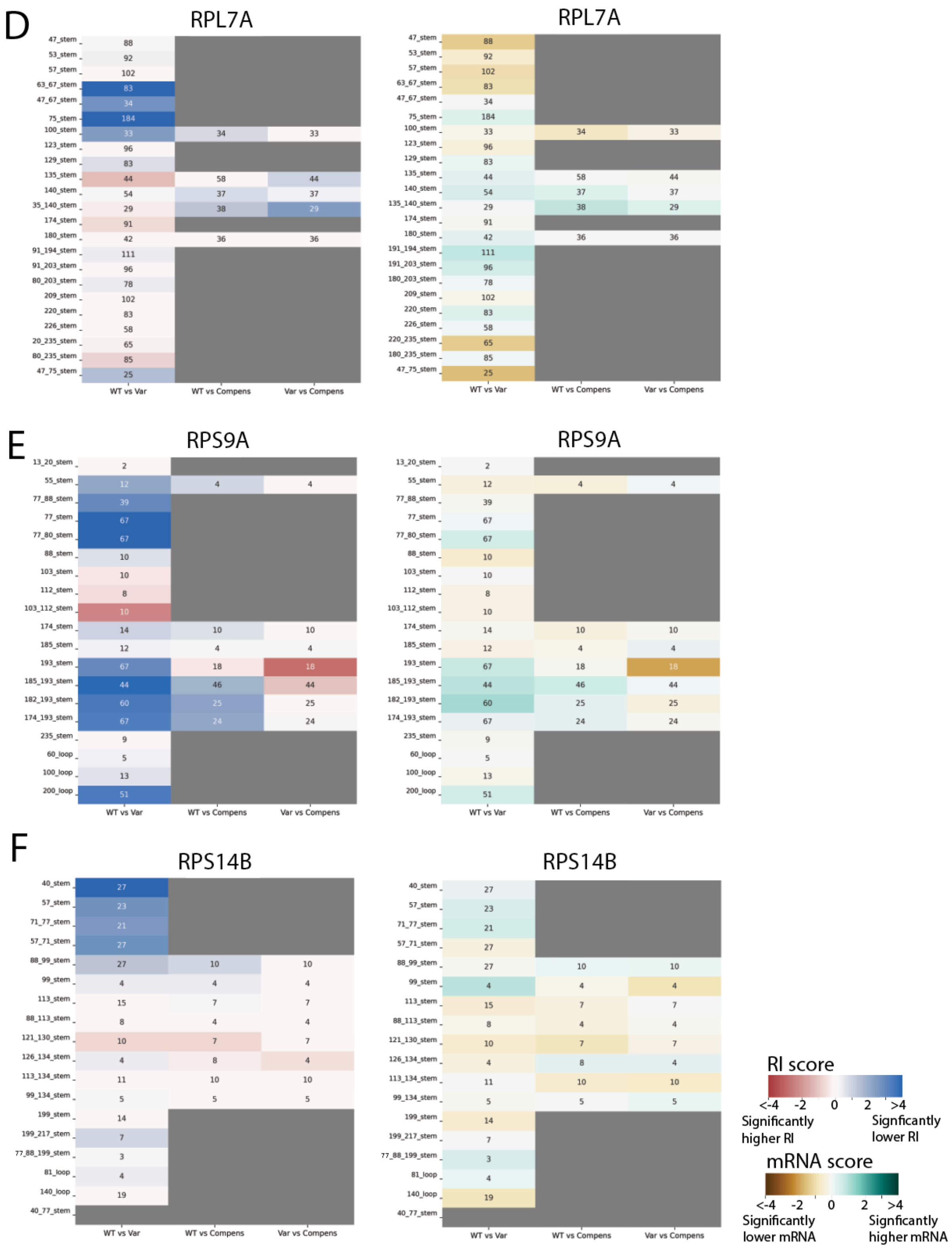
D
RPL7A
RPL7A
E
RPS9A
RPS9A
F
RPS14B
RPS14B
WT vs Var
WT vs Compens
Var vs Compens
RI score
<-4 -2 0 2 >4
Significantly higher RI
Signficantly lower RI
mRNA score
<-4 -2 0 2 >4
Significantly lower mRNA
Signficantly higher mRNA

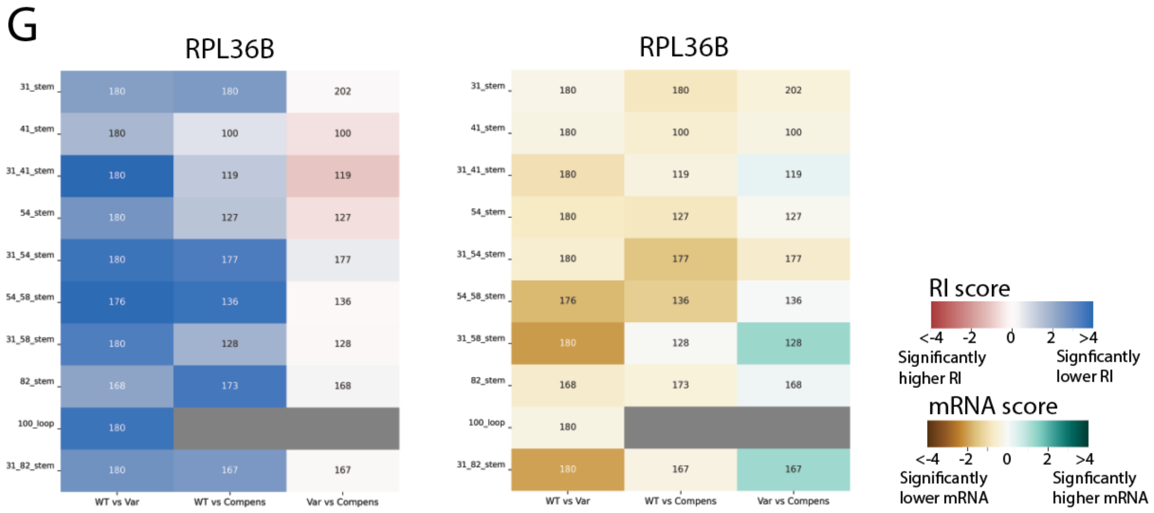

Supplementary Figure 16: Heatmaps depicting the effects of structure variants on retained intron fractions and normalized mRNA levels for **A)** *QCR9*, **B)** *RPL28*, **C)** *RPS9B*, **D)** *RPL7A*, **E)** *RPS9A*, **F)** *RPS14B*, and **G)** *RPL36B*. Each row corresponds to a stem or loop disruption set, and the nucleotides these labels correspond to are noted in Supplementary Table 3. Boxes are colored based on the significance of the difference in RI fraction or mRNA score between sequence sets, and numbers in boxes indicate the minimum number of sequences used for each comparison. Boxes are gray if comparisons involve 2 or fewer sequences, either due to low coverage or because the sequence set was not included in the library. The heatmap compares the following sets of sequences: 1) left column: wildtype sequences are the base set and variant sequences are the comparison set; 2) middle column: wildtype sequences are again the base set and compensatory rescue sequences are the comparison set; 3) right column: variant sequences are the base set and compensatory rescue sequences are the comparison set. The heatmaps are colored by the retained intron (RI) scores (left) and mRNA scores (right). The RI score is the negative log(p-value) comparing RI values between sets, and the sign indicates the effect direction, with positive values (shown as blue) for lower RI values in the comparison set compared to the base set, and negative values (shown as red) for higher RI values in the comparison set. Similarly, the mRNA score is computed as the negative log(p-value) comparing normalized mRNA levels between sets, with positive (green) values indicating higher comparison set mRNA levels, and negative (brown) values indicating lower comparison set mRNA levels. P-values are computed by two-sided permutation tests for the difference in mean statistic.

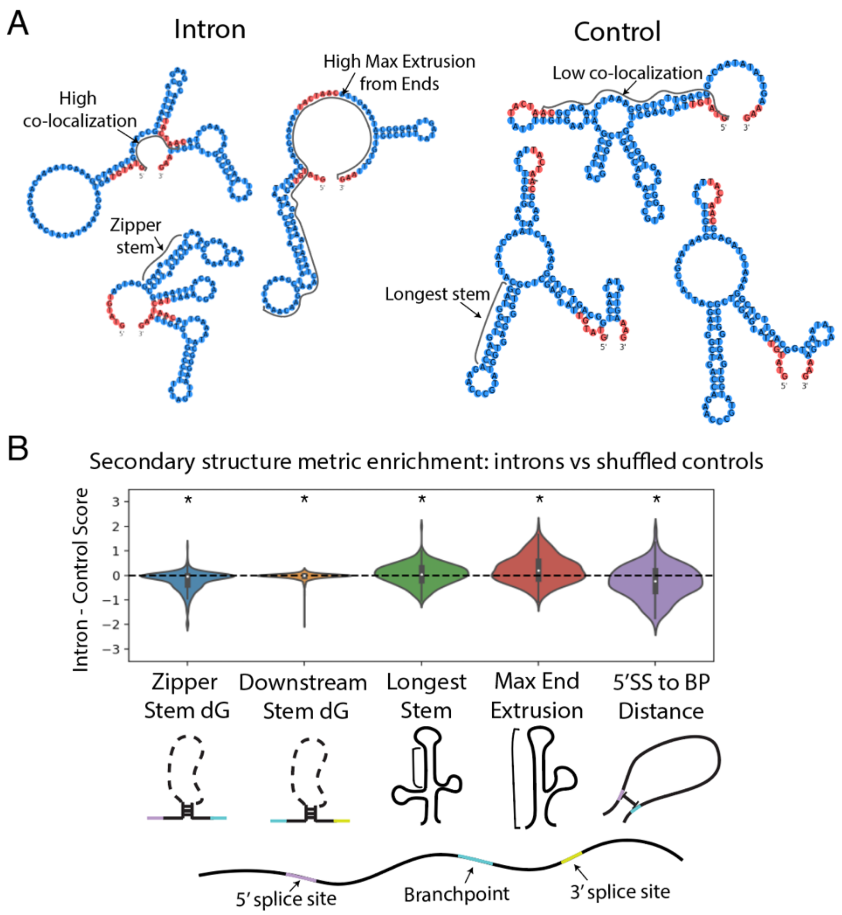

Supplementary Figure 17: Schematics for *de novo* secondary structure feature prediction. **A)** Sample structures from an intron secondary structure ensemble and control secondary structure ensemble, with structural features annotated. **B)** Enrichment of secondary structure features comparing intron sequences to shuffled sequences using secondary structure ensembles predicted from Vienna 2.0. *p-value < 0.01 by a two-sided Wilcoxon ranked-sum test with N=288 for all comparisons. From left to right, exact p-values are <1E-4, 0.00027, 0.0011, <1E-4, <1E-4. Box plots mark the median as the center white point and include a box from the 25th (Q1) to 75th (Q3) percentile, extending whiskers to the smallest and largest value that fall within 1.5 times the interquartile range below Q1 and above Q3.

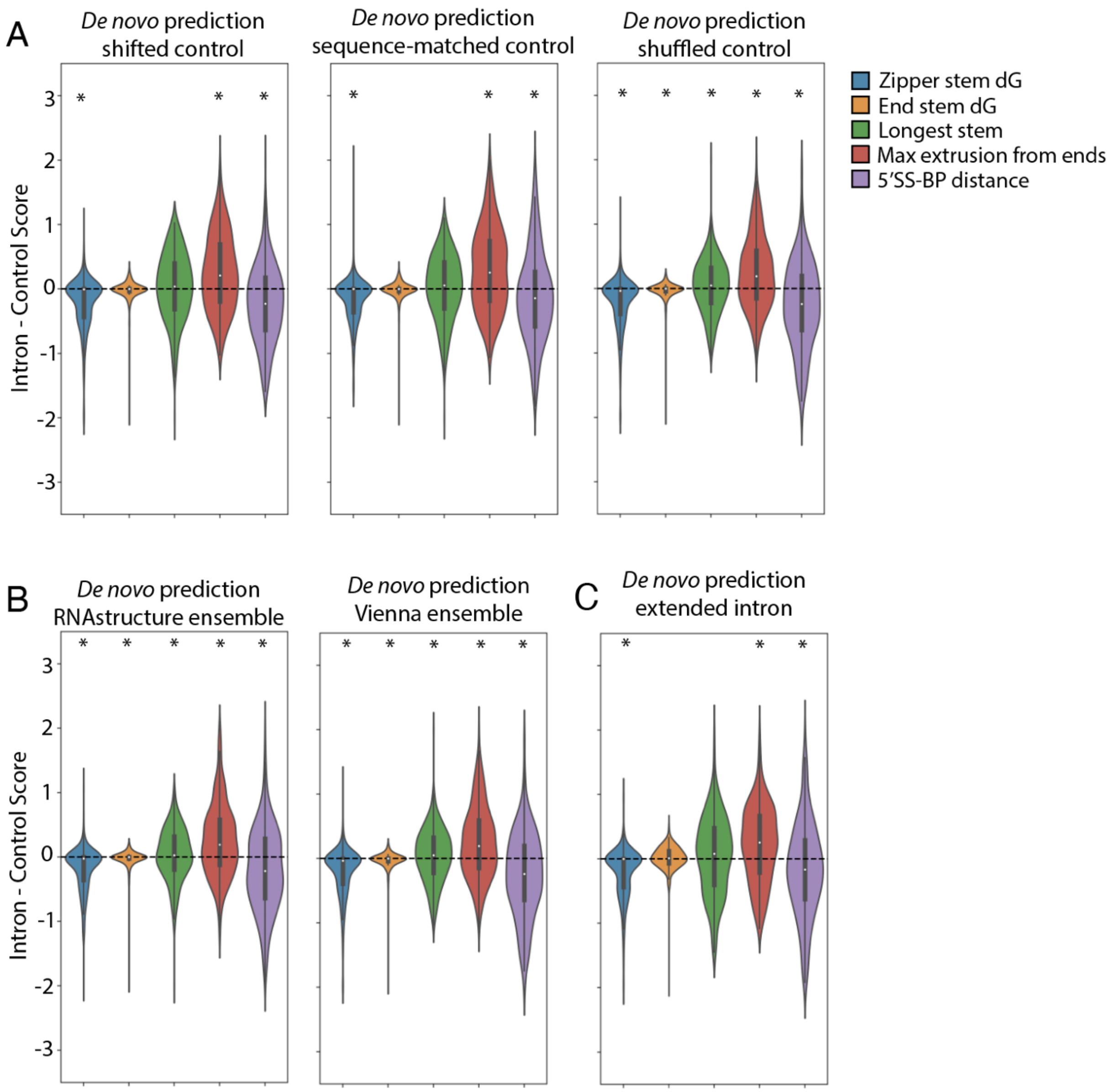

Supplementary Figure 18: Computational prediction of secondary structure feature enrichment with various parameters. **A)** Feature enrichment when comparing intron sequences to a various control sequence sets. **B)** Feature enrichment when predicting secondary structure ensembles with RNAstructure vs Vienna, comparing introns to shuffled controls. **C)** Feature enrichment when including a 50 nucleotide sequence context upstream and downstream of the intron. *p-value < 0.01 by a two-sided Wilcoxon ranked-sum test with N=288 for all comparisons. Box plots mark the median as the center white point and include a box from the 25th (Q1) to 75th (Q3) percentile, extending whiskers to the smallest and largest value that fall within 1.5 times the interquartile range below Q1 and above Q3. Exact p-values are as follows from left to right for each plot: A) left: <1E-4, 0.060, 0.034, <1E-4, <1E-4; A) middle: <1E-4, 0.055, 0.031, <1E-4, 0.0017; A) right: <1E-4, 0.00027, 0.0011, <1E-4, <1E-4; B) left: <1E-4, 0.0054, 0.0026, <1E-4, <1E-4; B) middle: <1E-4, 0.00027, 0.0011, <1E-4, <1E-4; C): <1E-4, 0.021, 0.091, <1E-4, <1E-4.

Supplementary Figure 19: Intron secondary structure properties across *Saccharomyces* genus. **A)** P-values (log scale, base 10) as computed by Wilcoxon ranked sum test comparing secondary structure metrics for intron and shuffled sequence control ensembles across *Saccharomyces* yeast species. **B)** P-values computed by two-sided Wilcoxon ranked sum test for secondary structure metric comparisons between introns and phylogenetic control sequences (log scale, base 10). **C)** Statistics on the number of orthologs for each *S. cerevisiae* intron and zipper stem across the *Saccharomyces* genus. **D)** Statistics on sequence conservation for introns (full sequence and zipper stem sequences specifically) across the genus. The species analyzed in this figure are as follows, with label abbreviations noted: *E. gossypii* (agos), *C. glabrata* (cgla), *E. cymbalariae* (ecym), *K. africana* (kafr), *K. lactis* (klac), *K. naganishii* (knag), *V. polyspora* (kpol), *L. thermotolerans* (kthe), *L. waltii* (kwal), *N. castellii* (ncas), *N. dairenensis* (ndai), *S. kudiavzevii* (skud), *S. mikatae* (smik), *S. uvarum* (suva), *T. blattae* (tbla), *T. delbrueckii* (tdel), *T. phaffii* (tpha), *Z. rouxii* (zrou).

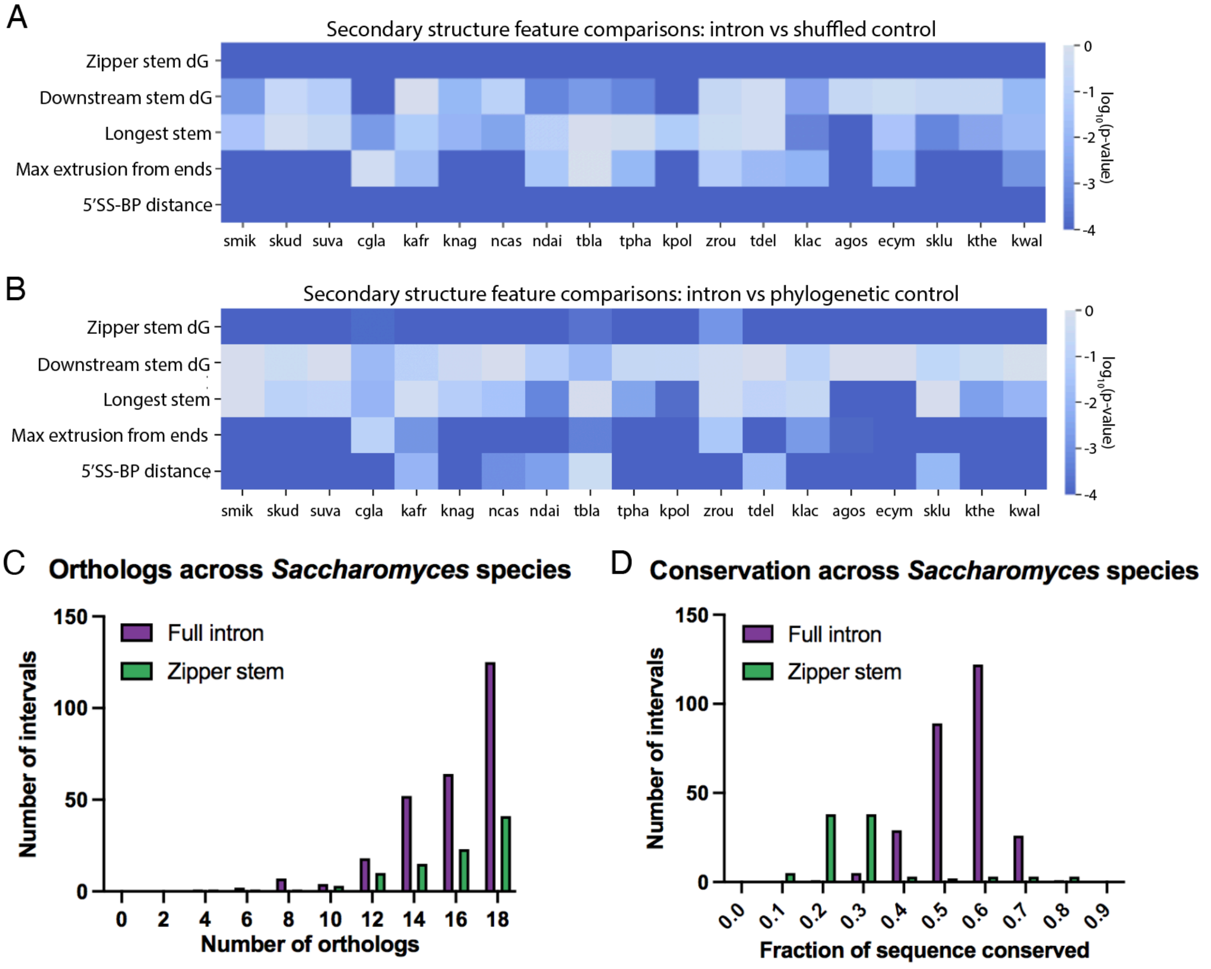

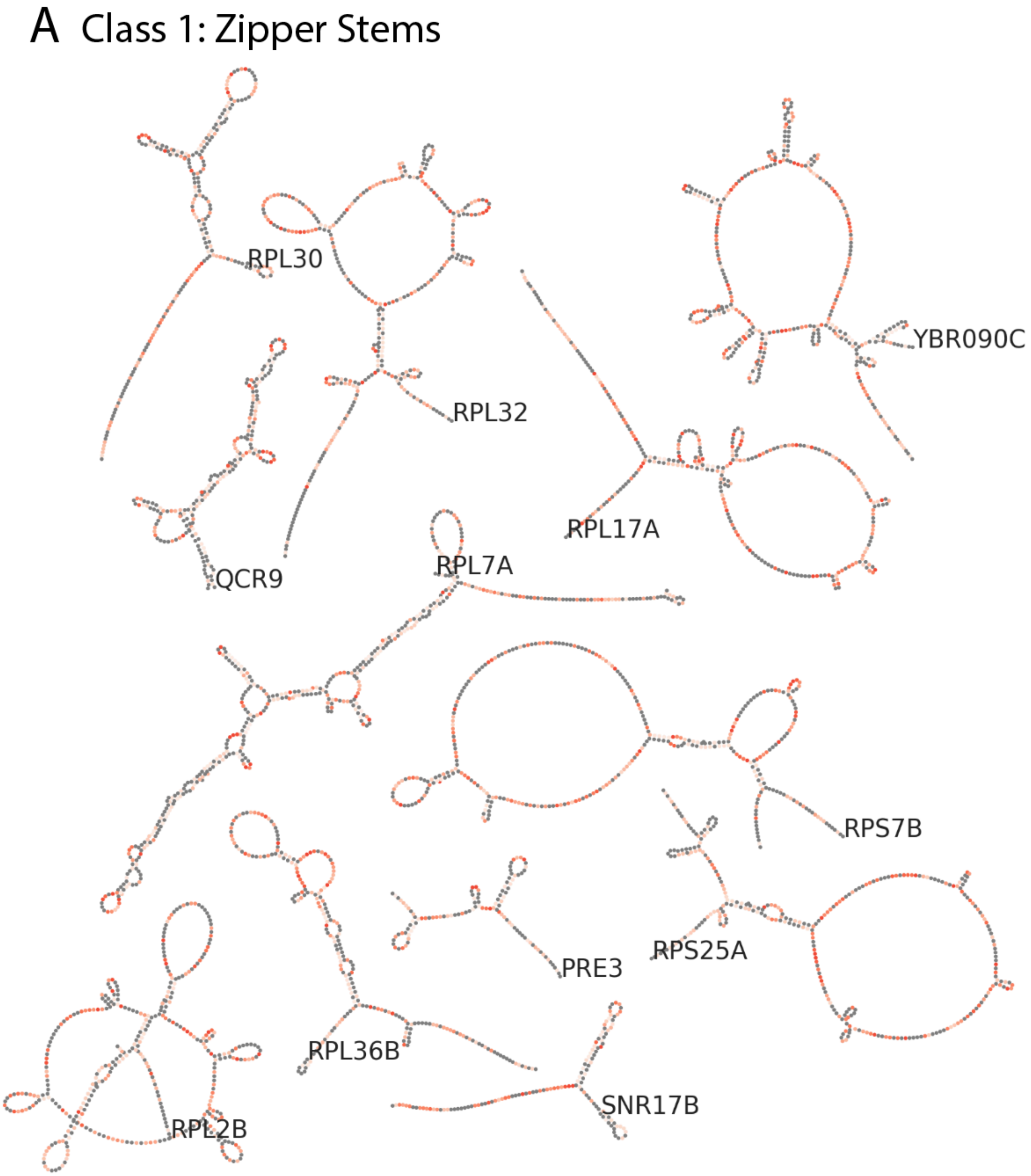
A
Class 1: Zipper Stems
RPL30
YBR090C
RPL32
QCR9
RPL17A
RPL7A
RPS7B
PRE3
RPS25A
RPL36B
RPL2B
SNR17B

B Class 1: Zipper Stems, continued

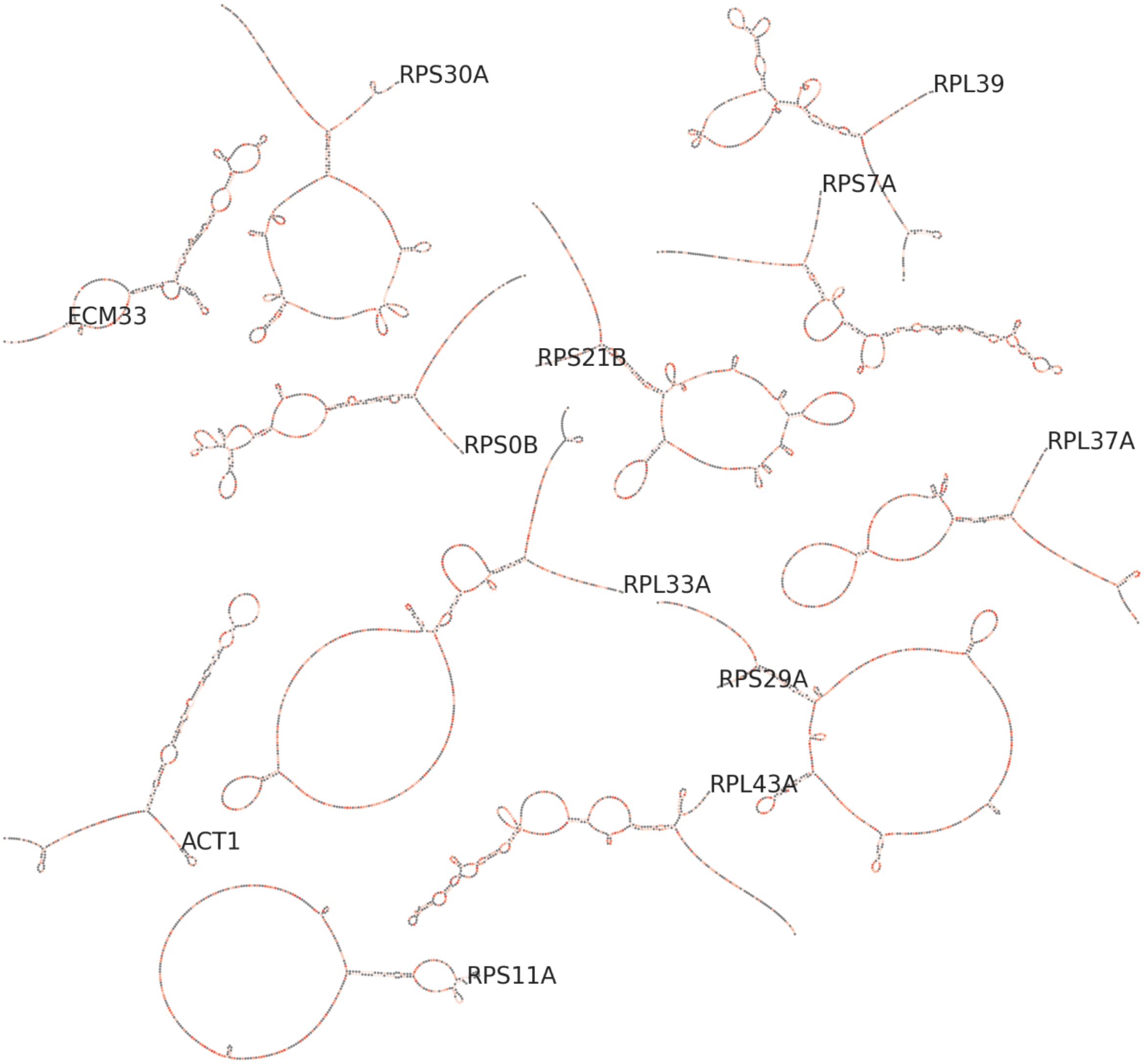

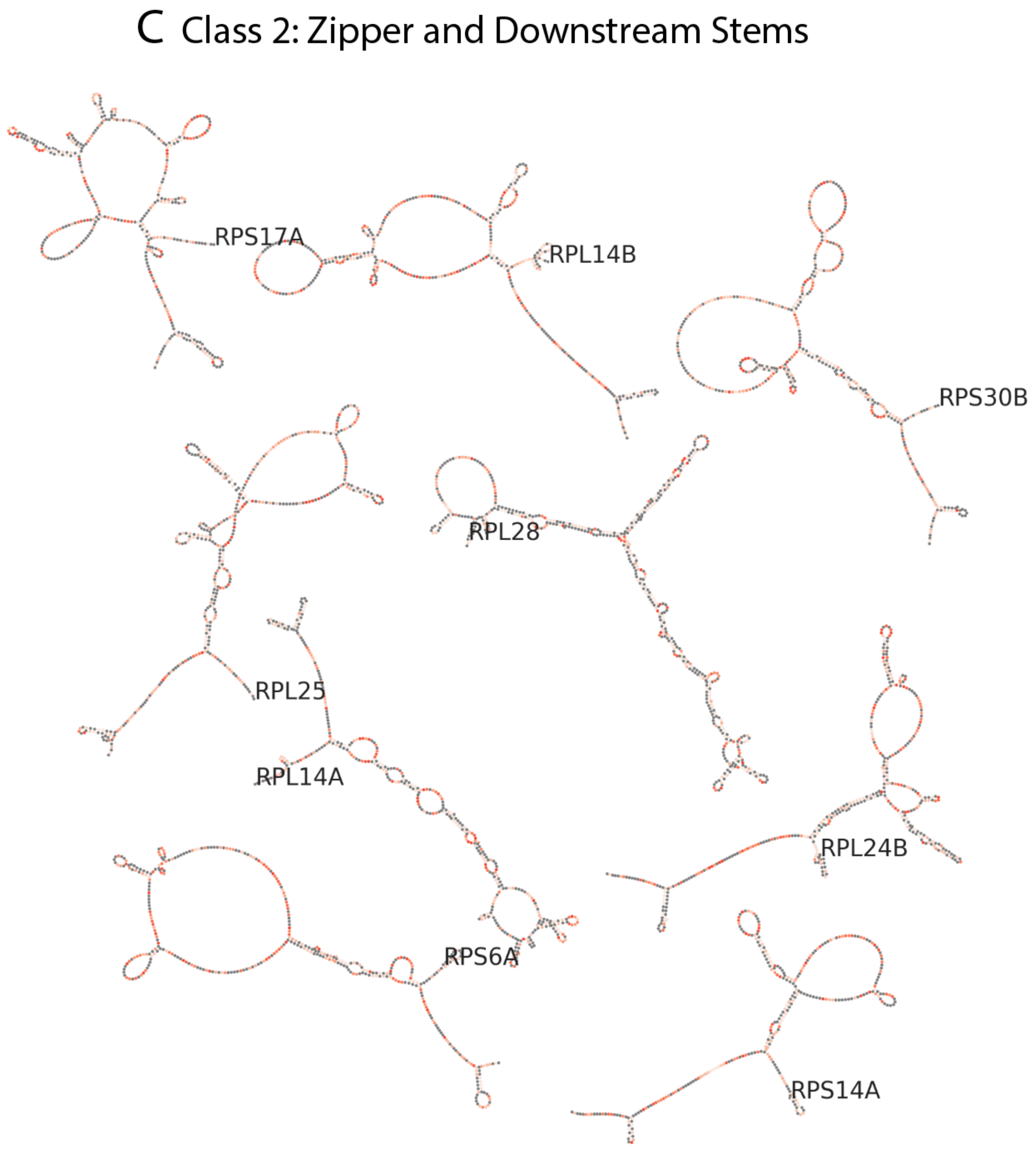
C Class 2: Zipper and Downstream Stems
RPS17A
RPL14B
RPS30B
RPL28
RPL25
RPL14A
RPL24B
RPS6A
RPS14A

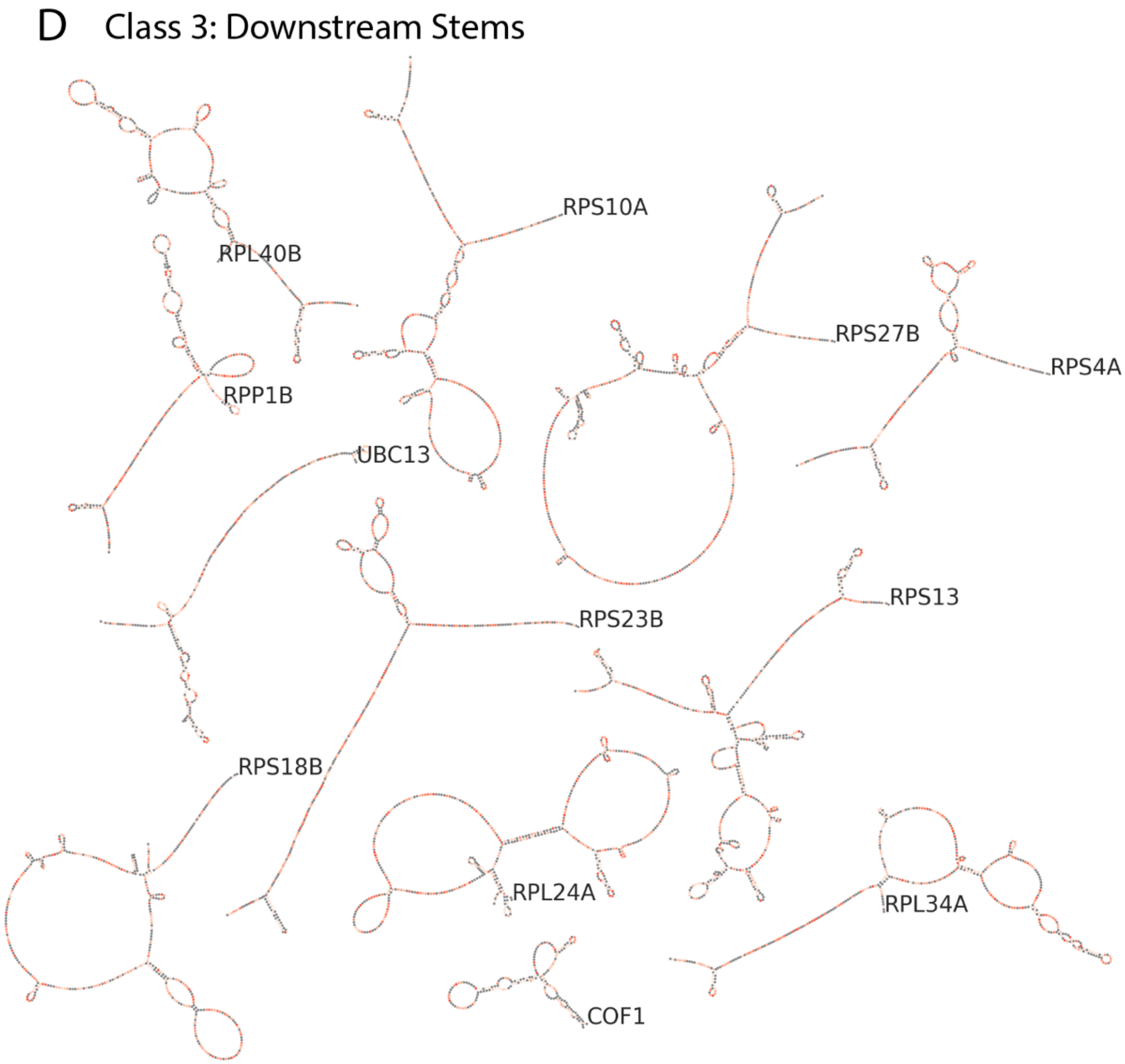
D
Class 3: Downstream Stems
RPS10A
RPL40B
RPS27B
RPS4A
RPP1B
UBC13
RPS13
RPS23B
RPS18B
RPL24A
RPL34A
COF1

E Class 4: Remaining Structured Long Introns

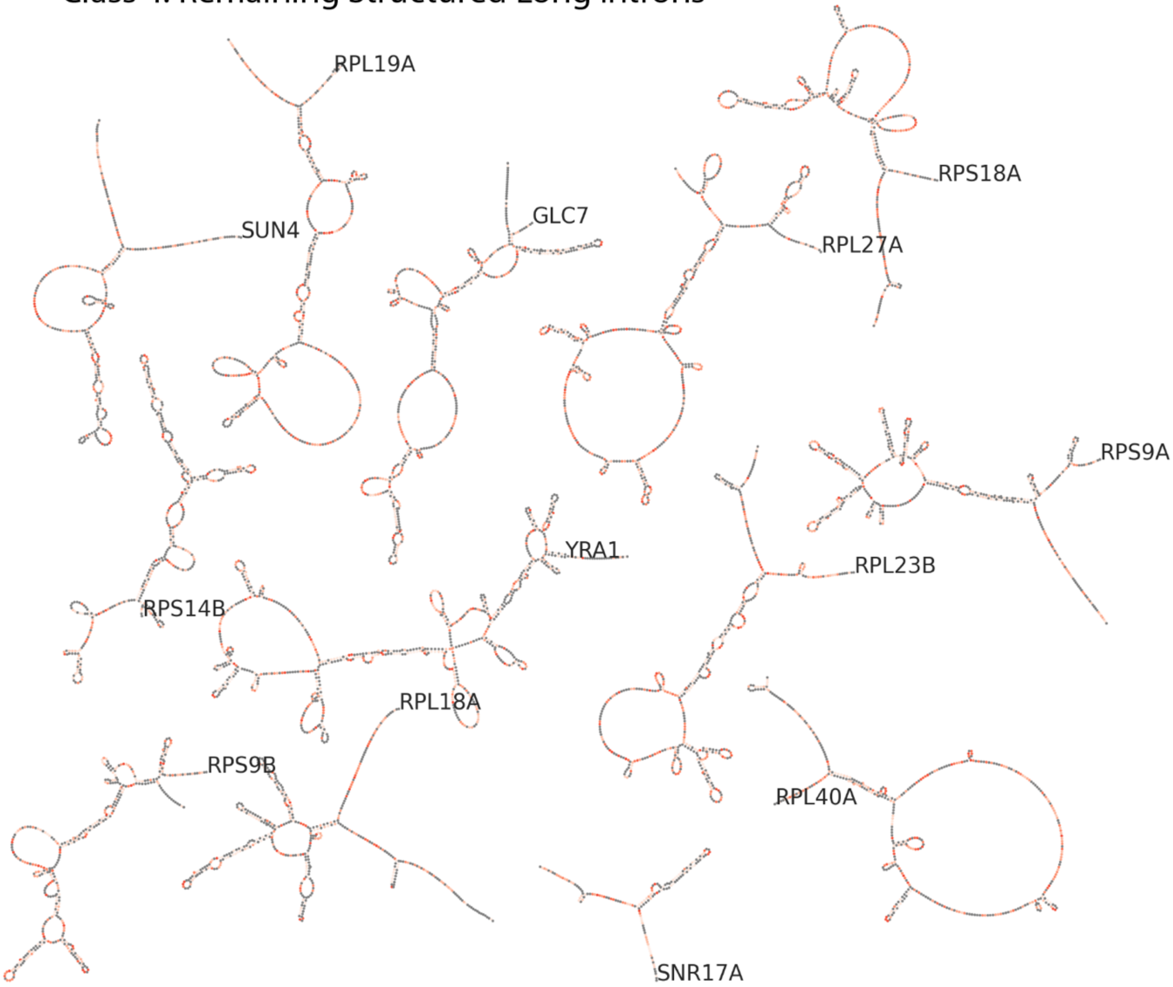

F Class 5: Long Introns with Low or Intermediate Structure

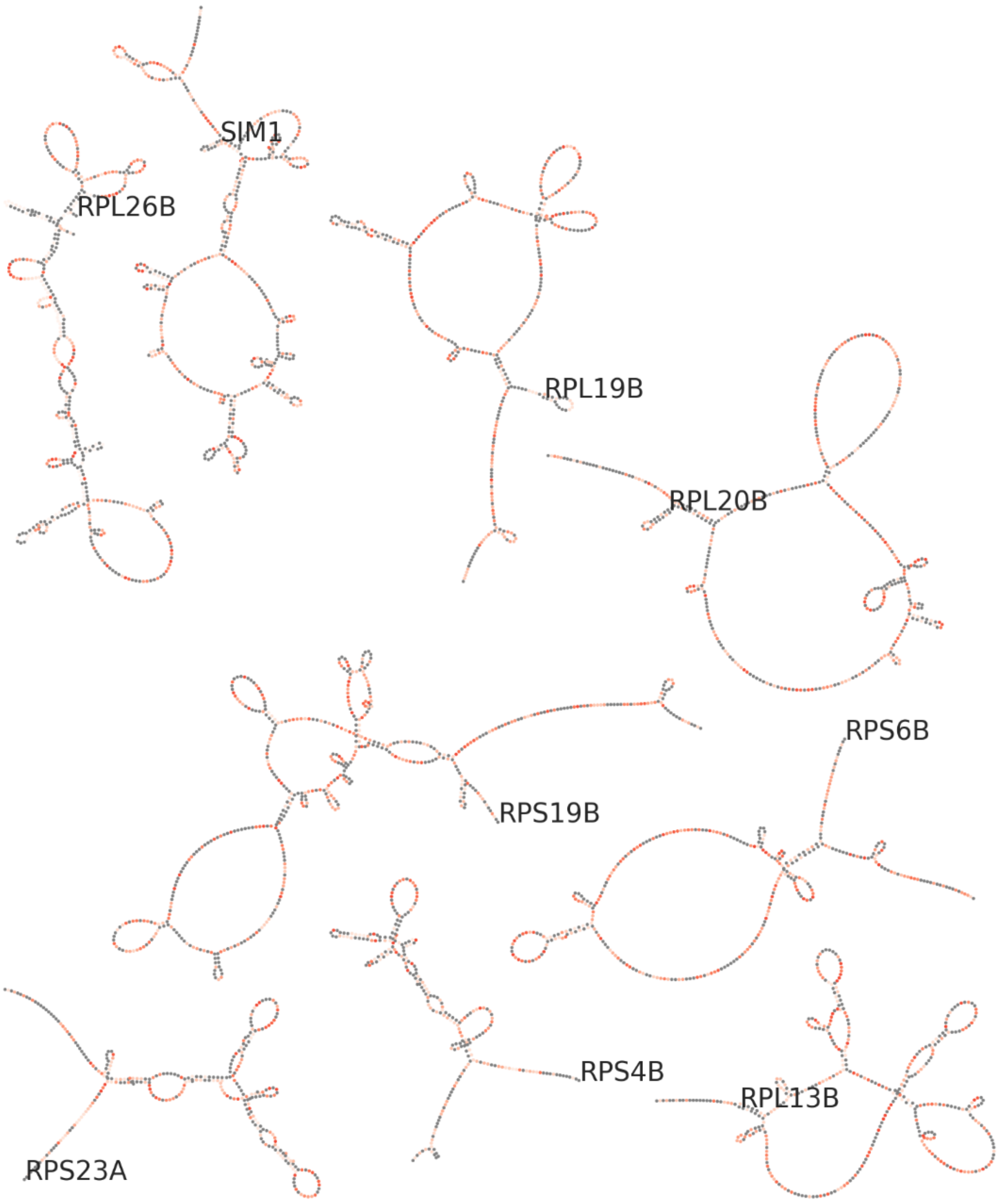

G Class 5: Long Introns with Low or Intermediate Structure, continued

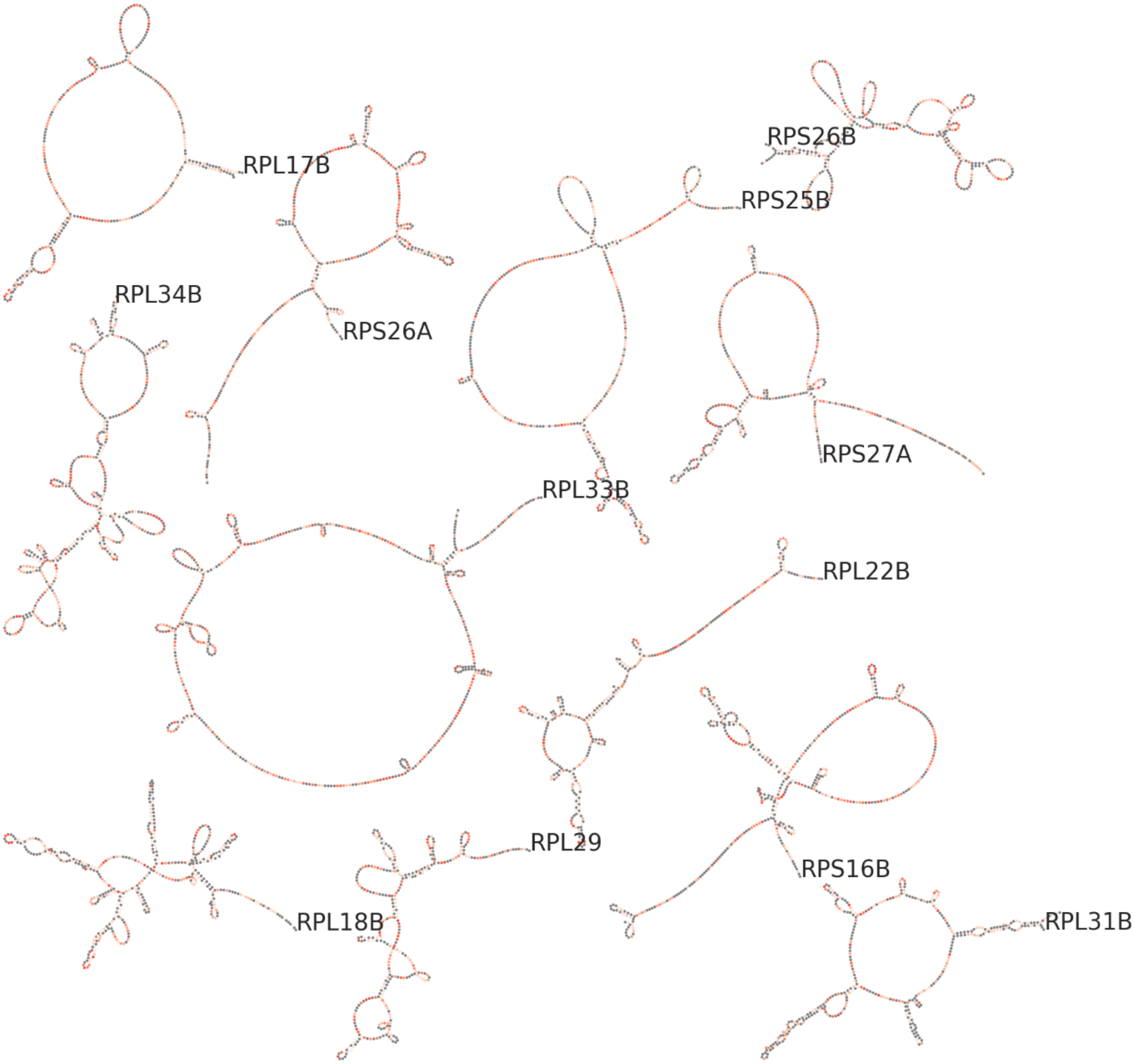

H Class 6: Unstructured Short Introns

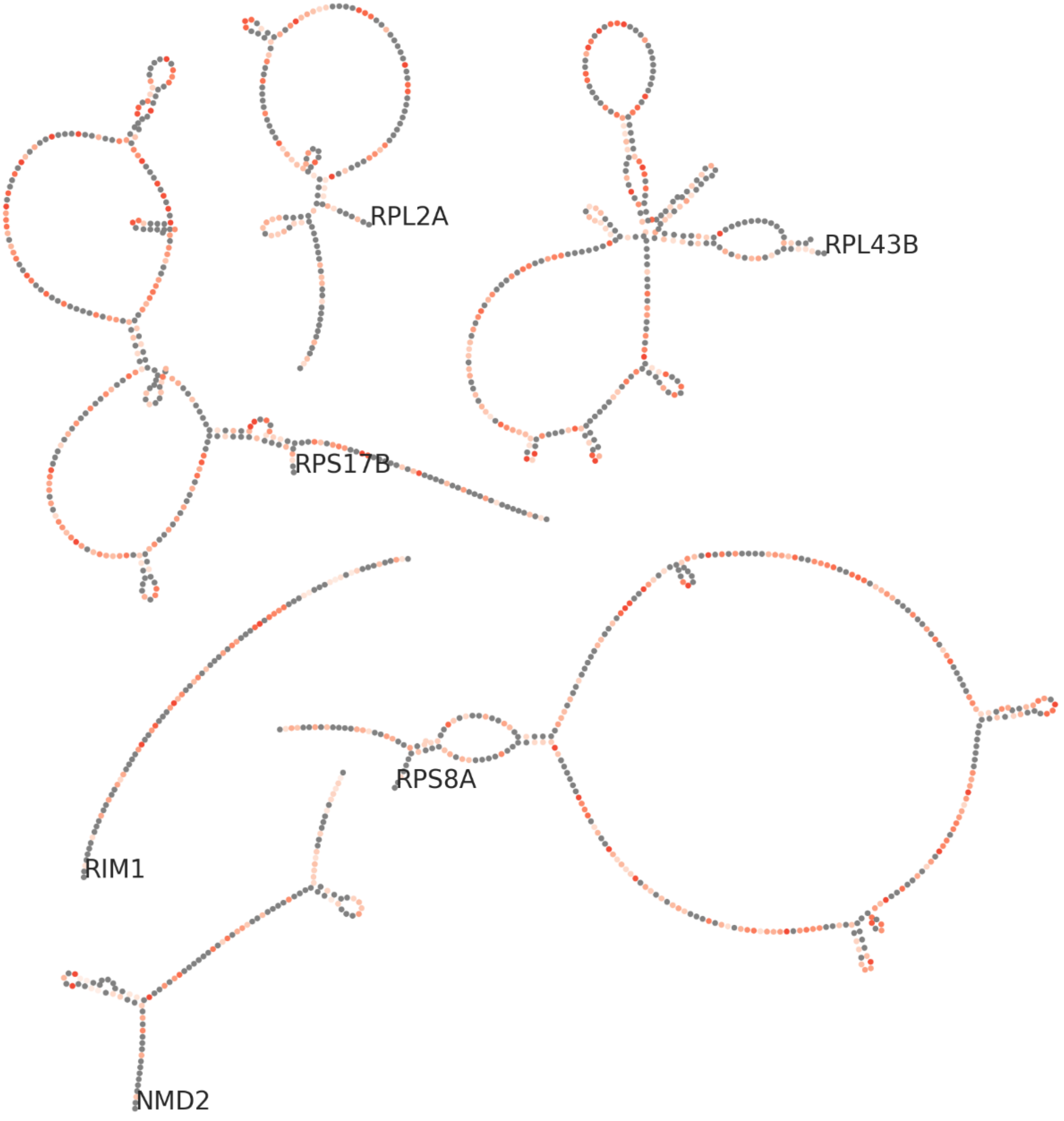

I Class 7: Structured Short Introns

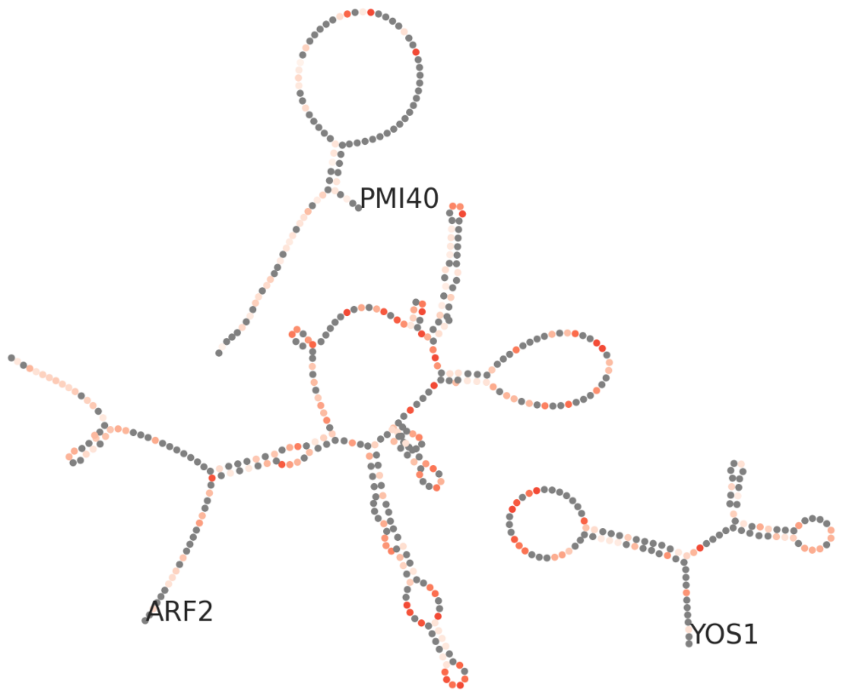

Supplementary Figure 20: Secondary structures and overlaid DMS reactivity profiles for all introns with sufficient coverage from DMS-MaPseq. Introns are grouped into classes from hierarchical clustering (Fig. 5). Secondary structures are depicted schematically using RiboGraphViz (https://github.com/DasLab/RiboGraphViz). Gene names are included for each intron, with all labels at the 5’ ends of introns.

Gene name	Class	Length	TP	FP	FN
RDN18-1 (18S rRNA)	rRNA	1800	14	4	15
RDN5-1 (5S rRNA)	rRNA	121	4	0	0
RDN58-1 (5.8S rRNA)	rRNA	158	1	0	0
SNR7-L (U5 snRNA)	snRNA	214	4	3	2
SNR19 (U1 snRNA)	snRNA	568	18	2	4
TRR4	tRNA	72	1	0	2
TRT2	tRNA	72	2	0	1
IMT4	tRNA	72	2	0	1
HAC1 mRNA	mRNA	382	1	1	0
ASH1 mRNA	mRNA	356	2	0	0
RPS28B mRNA	mRNA	351	2	0	0
SFT2 mRNA	mRNA	301	0	0	1
Total (mRNA)	Total	--	5	1	0
Total	Total	--	51	11	26

Supplementary Table 2: True positive, false positive, and false negative stem predictions across a set of positive control structures including rRNAs, snRNAs, tRNAs, and mRNA segments. Secondary structure predictions are made using a 70% helix confidence estimate cutoff from bootstrapping DMS reactivity values. Stems are included if they include at least 5 base-pairs. The ground truth secondary structures for the 5S, 5.8S, and 18S rRNA were obtained from a high-resolution X-ray crystallography structure of the eukaryotic ribosome (PDB ID: 4V88)[20]. Structures for the U5 snRNA and U1 snRNA were obtained from Nguyen, et. al. (2016)[21] and Li, et. al. (2017)[22] respectively. Rfam-derived secondary structures[23] served as ground truth structures for the four tRNA structures, and structures for mRNA segments in *HAC1*, *ASH1*, *RPS28B*, and *SFT2* were obtained from Zubradt, et al. (2017)[24].

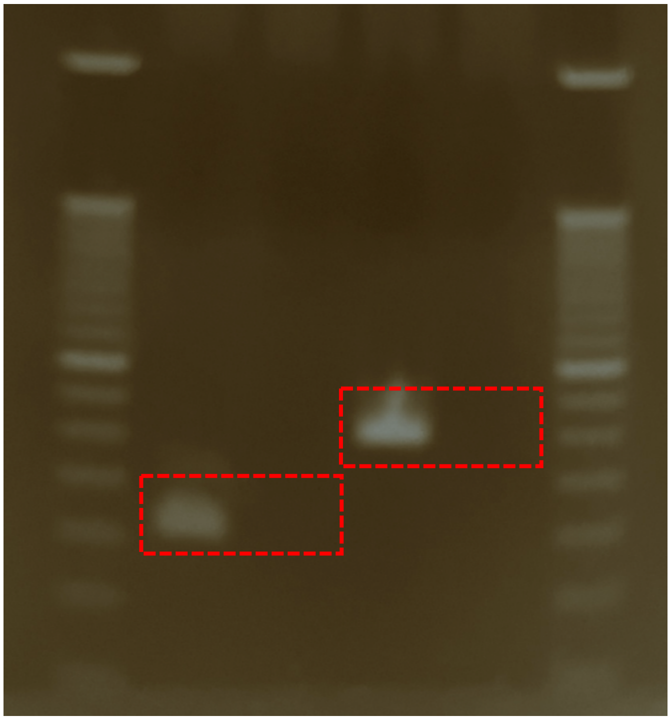

Source Data for Supplementary Figure 13: Uncropped gel from Supplementary Figure 13C. RT-PCR for control regions (RNA intervals in *RPL36B* and *MATa1*) demonstrating depletion of gDNA from targeted RNA-sequencing library preparation.

References

1 Hajdin, C. E. *et al.* Accurate SHAPE-directed RNA secondary structure modeling, including pseudoknots. *Proc Natl Acad Sci U S A* **110**, 5498-5503 (2013). https://doi.org:10.1073/pnas.1219988110

2 Lan, P. *et al.* Structural insight into precursor tRNA processing by yeast ribonuclease P. *Science* **362** (2018). https://doi.org:10.1126/science.aat6678

3 Tomezsko, P. J. *et al.* Determination of RNA structural diversity and its role in HIV-1 RNA splicing. *Nature* **582**, 438-442 (2020). https://doi.org:10.1038/s41586-020-2253-5

4 Charpentier, B. & Rosbash, M. Intramolecular structure in yeast introns aids early steps of in vitro spliceosome assembly. *RNA* (1996).

5 Rogic, S. *et al.* Correlation between the secondary structure of pre-mRNA introns and the efficiency of splicing in Saccharomyces cerevisiae. *BMC Genomics* **9**, 355 (2008). https://doi.org:10.1186/1471-2164-9-355

6 Meyer, M., Plass, M., Perez-Valle, J., Eyras, E. & Vilardell, J. Deciphering 3'ss selection in the yeast genome reveals an RNA thermosensor that mediates alternative splicing. *Mol Cell* **43**, 1033-1039 (2011). https://doi.org:10.1016/j.molcel.2011.07.030

7 Vilardell, J. & Warner, J. R. Regulation of splicing at an intermediate step in the formation of the spliceosome. *Genes Dev* **8**, 211-220 (1994). https://doi.org:10.1101/gad.8.2.211

8 Li, Z., Paulovich, A. G. & Woolford, J. L., Jr. Feedback inhibition of the yeast ribosomal protein gene CRY2 is mediated by the nucleotide sequence and secondary structure of CRY2 pre-mRNA. *Mol Cell Biol* **15**, 6454-6464 (1995). https://doi.org:10.1128/mcb.15.11.6454

9 Plocik, A. M. & Guthrie, C. Diverse forms of RPS9 splicing are part of an evolving autoregulatory circuit. *PLoS Genet* **8**, e1002620 (2012). https://doi.org:10.1371/journal.pgen.1002620

10 Fewell, S. W. & Woolford, J. L., Jr. Ribosomal protein S14 of Saccharomyces cerevisiae regulates its expression by binding to RPS14B pre-mRNA and to 18S rRNA. *Mol Cell Biol* **19**, 826-834 (1999). https://doi.org:10.1128/mcb.19.1.826

11 Danin-Kreiselman, M., Lee, C. Y. & Chanfreau, G. RNAse III-Mediated Degradation of Unspliced Pre-mRNAs and Lariat Introns. *Molecular Cell* **11**, 1279-1289 (2003). https://doi.org:10.1016/s1097-2765(03)00137-0

12 Hooks, K. B., Naseeb, S., Parker, S., Griffiths-Jones, S. & Delneri, D. Novel Intronic RNA Structures Contribute to Maintenance of Phenotype in Saccharomyces cerevisiae. *Genetics* **203**, 1469-1481 (2016). https://doi.org:10.1534/genetics.115.185363

13 Yao, Z., Weinberg, Z. & Ruzzo, W. L. CMfinder--a covariance model based RNA motif finding algorithm. *Bioinformatics* **22**, 445-452 (2006). https://doi.org:10.1093/bioinformatics/btk008

14 Gruber, A. R., Neubock, R., Hofacker, I. L. & Washietl, S. The RNAz web server: prediction of thermodynamically stable and evolutionarily conserved RNA structures. *Nucleic Acids Res* **35**, W335-338 (2007). https://doi.org:10.1093/nar/gkm222

15 Pedersen, J. S. *et al.* Identification and classification of conserved RNA secondary structures in the human genome. *PLoS Comput Biol* **2**, e33 (2006). https://doi.org:10.1371/journal.pcbi.0020033

16 Gao, W., Jones, T. A. & Rivas, E. Discovery of 17 conserved structural RNAs in fungi. *Nucleic Acids Res* **49**, 6128-6143 (2021). https://doi.org:10.1093/nar/gkab355
17 Rivas, E., Clements, J. & Eddy, S. R. A statistical test for conserved RNA structure shows lack of evidence for structure in lncRNAs. *Nat Methods* **14**, 45-48 (2017). https://doi.org:10.1038/nmeth.4066
18 Cheng, C. Y., Kladwang, W., Yesselman, J. D. & Das, R. RNA structure inference through chemical mapping after accidental or intentional mutations. *Proc Natl Acad Sci U S A* **114**, 9876-9881 (2017). https://doi.org:10.1073/pnas.1619897114
19 Lee, M. E., DeLoache, W. C., Cervantes, B. & Dueber, J. E. A Highly Characterized Yeast Toolkit for Modular, Multipart Assembly. *ACS Synth Biol* **4**, 975-986 (2015). https://doi.org:10.1021/sb500366v
20 Ben-Shem, A. *et al.* The structure of the eukaryotic ribosome at 3.0 A resolution. *Science* **334**, 1524-1529 (2011). https://doi.org:10.1126/science.1212642
21 Nguyen, T. H. D. *et al.* Cryo-EM structure of the yeast U4/U6.U5 tri-snRNP at 3.7 A resolution. *Nature* **530**, 298-302 (2016). https://doi.org:10.1038/nature16940
22 Li, X. *et al.* CryoEM structure of Saccharomyces cerevisiae U1 snRNP offers insight into alternative splicing. *Nat Commun* **8**, 1035 (2017). https://doi.org:10.1038/s41467-017-01241-9
23 Kalvari, I. *et al.* Rfam 14: expanded coverage of metagenomic, viral and microRNA families. *Nucleic Acids Res* **49**, D192-D200 (2021). https://doi.org:10.1093/nar/gkaa1047
24 Zubradt, M. *et al.* DMS-MaPseq for genome-wide or targeted RNA structure probing in vivo. *Nat Methods* **14**, 75-82 (2017). https://doi.org:10.1038/nmeth.4057
