# Supplementary material for: Comprehensive analysis of Saccharomyces cerevisiae intron structures in vivo
Source: Nat Struct Mol Biol. 2025 Jun 5;32(8):1488–502. doi: 10.1038/s41594-025-01565-x (PMC12350175; doi:10.1038/s41594-025-01565-x)
Supplement: Supplementary file 2 — Reporting Summary [file 41594_2025_1565_MOESM2_ESM.pdf]

nature portfolio

Corresponding author(s): Rhiju Das

Last updated by author(s): Mar 16, 2025

Reporting Summary

Nature Portfolio wishes to improve the reproducibility of the work that we publish. This form provides structure for consistency and transparency in reporting. For further information on Nature Portfolio policies, see our Editorial Policies and the Editorial Policy Checklist.

Statistics

For all statistical analyses, confirm that the following items are present in the figure legend, table legend, main text, or Methods section.

n/a	Confirmed	
☐	☒	The exact sample size (*n*) for each experimental group/condition, given as a discrete number and unit of measurement
☒	☐	A statement on whether measurements were taken from distinct samples or whether the same sample was measured repeatedly
☐	☒	The statistical test(s) used AND whether they are one- or two-sided<br>*Only common tests should be described solely by name; describe more complex techniques in the Methods section.*
☒	☐	A description of all covariates tested
☒	☐	A description of any assumptions or corrections, such as tests of normality and adjustment for multiple comparisons
☐	☒	A full description of the statistical parameters including central tendency (e.g. means) or other basic estimates (e.g. regression coefficient) AND variation (e.g. standard deviation) or associated estimates of uncertainty (e.g. confidence intervals)
☐	☒	For null hypothesis testing, the test statistic (e.g. *F*, *t*, *r*) with confidence intervals, effect sizes, degrees of freedom and *P* value noted<br>*Give P values as exact values whenever suitable.*
☒	☐	For Bayesian analysis, information on the choice of priors and Markov chain Monte Carlo settings
☒	☐	For hierarchical and complex designs, identification of the appropriate level for tests and full reporting of outcomes
☐	☒	Estimates of effect sizes (e.g. Cohen's *d*, Pearson's *r*), indicating how they were calculated

Our web collection on statistics for biologists contains articles on many of the points above.

Software and code

Policy information about availability of computer code

Data collection	No software was used for data collection.
Data analysis	Sequencing data processing pipelines are detailed at the GEO accession GSE209857. The following software was used for sequencing data analysis: bowtie2 (v2.3.4.1), cutadapt (v1.18), UMI-tools (v1.1.1), samtools (v1.16.1), fgbio (v2.0.2), gmap (2015-12-31), TopHat (v2.1.1), Biers (v1.2), RNAframework (v2.6.9), and ShapeMapper (v1.2). The following tools were used for structure prediction and structure analysis: Rosetta (v3.5), RNAstructure (v6.1), Vienna (v2.4.13), Contrafold (v2.00), DREEM (v0.0.2), Arnie (v0.2.6), and R-scape (v1.6.0). RiboDraw (v1.0.0) and RiboGraphViz (https://github.com/DasLab/RiboGraphViz) were used for structure visualization. Structural feature calculation, statistical comparisons, and evolutionary analyses are included at https://github.com/ramyarangan/pre-mRNA_Secstruct. Code for designing VARS-seq sequences and analyzing gDNA and RNA sequencing data from VARS-seq is included in https://github.com/ramyarangan/VARSseq. Analysis of intron evolution and computational predictions for intron secondary structures and structural features were performed using the code in: https://github.com/ramyarangan/pre-mRNA_Secstruct.

For manuscripts utilizing custom algorithms or software that are central to the research but not yet described in published literature, software must be made available to editors and reviewers. We strongly encourage code deposition in a community repository (e.g. GitHub). See the Nature Portfolio guidelines for submitting code & software for further information.

Data

Policy information about availability of data

All manuscripts must include a data availability statement. This statement should provide the following information, where applicable:

- Accession codes, unique identifiers, or web links for publicly available datasets
- A description of any restrictions on data availability
- For clinical datasets or third party data, please ensure that the statement adheres to our policy

All sequencing data are available at the Gene Expression Omnibus (GEO) accession number GSE209857. DMS-derived secondary structures are included in Table S1. Processed DMS reactivity data, control RNA secondary structures, and base-pairing probabilities from structure prediction with bootstrapping are included in https://github.com/ramyarangan/DMS_intron_analysis. VARS-seq intron variant sequences along with spliced and unspliced read counts are included in Tables S3-S4. PDB IDs 6G90, 4V88, 6AGB, and 6N7R were used for analysis. We compared our data with prior DMS-MaPseq data included at GEO accession GSE84537 (sample SRX1959209, run number SRR3929621). We additionally used annotations from the Saccharomyces Genome Database, S. cerevisiae snoRNA database, and the YeasTSS database.

Research involving human participants, their data, or biological material

Policy information about studies with human participants or human data. See also policy information about sex, gender (identity/presentation), and sexual orientation and race, ethnicity and racism.

Reporting on sex and gender	N/A
Reporting on race, ethnicity, or other socially relevant groupings	N/A
Population characteristics	N/A
Recruitment	N/A
Ethics oversight	N/A

Note that full information on the approval of the study protocol must also be provided in the manuscript.

Field-specific reporting

Please select the one below that is the best fit for your research. If you are not sure, read the appropriate sections before making your selection.

☒ Life sciences ☐ Behavioural & social sciences ☐ Ecological, evolutionary & environmental sciences

For a reference copy of the document with all sections, see nature.com/documents/nr-reporting-summary-flat.pdf

Life sciences study design

All studies must disclose on these points even when the disclosure is negative.

Sample size	Structure probing experiments were carried out in two replicates per condition, aligning with prior standards in the field.
Data exclusions	No data were excluded.
Replication	For all transcriptome-wide structure probing experiments (in vivo, in vitro refolded, and denatured conditions) along with targeted DMS-MaPseq, we performed experiments with two replicates to confirm replicable structure profiles. Biological replicates were additionally performed for RT-qPCR experiments. In all cases, replicate experiments were performed by growing overnight cultures from independent yeast clones, with separate cell treatment and RNA extraction. All attempts at replicating experiments were performed successfully.
Randomization	Randomization was not relevant to this study design, as experiments were conducted where treatments were applied to some sequences but not others.
Blinding	Blinding was not relevant to this study design, as we did not expect that bias would influence outcomes of experiments.

Reporting for specific materials, systems and methods

We require information from authors about some types of materials, experimental systems and methods used in many studies. Here, indicate whether each material, system or method listed is relevant to your study. If you are not sure if a list item applies to your research, read the appropriate section before selecting a response.

Materials & experimental systems

n/a	Involved in the study
☒	☐ Antibodies
☒	☐ Eukaryotic cell lines
☒	☐ Palaeontology and archaeology
☒	☐ Animals and other organisms
☒	☐ Clinical data
☒	☐ Dual use research of concern
☒	☐ Plants

Methods

n/a	Involved in the study
☒	☐ ChIP-seq
☒	☐ Flow cytometry
☒	☐ MRI-based neuroimaging

Plants

Seed stocks

Report on the source of all seed stocks or other plant material used. If applicable, state the seed stock centre and catalogue number. If plant specimens were collected from the field, describe the collection location, date and sampling procedures.

Novel plant genotypes

Describe the methods by which all novel plant genotypes were produced. This includes those generated by transgenic approaches, gene editing, chemical/radiation-based mutagenesis and hybridization. For transgenic lines, describe the transformation method, the number of independent lines analyzed and the generation upon which experiments were performed. For gene-edited lines, describe the editor used, the endogenous sequence targeted for editing, the targeting guide RNA sequence (if applicable) and how the editor was applied.

Authentication

Describe any authentication procedures for each seed stock used or novel genotype generated. Describe any experiments used to assess the effect of a mutation and, where applicable, how potential secondary effects (e.g. second site T-DNA insertions, mosiacism, off-target gene editing) were examined.
