# Supplementary figures and images for: Comprehensive analysis of Saccharomyces cerevisiae intron structures in vivo
Source: Nat Struct Mol Biol. 2025 Jun 5;32(8):1488–502. doi: 10.1038/s41594-025-01565-x (PMC12350175; doi:10.1038/s41594-025-01565-x)

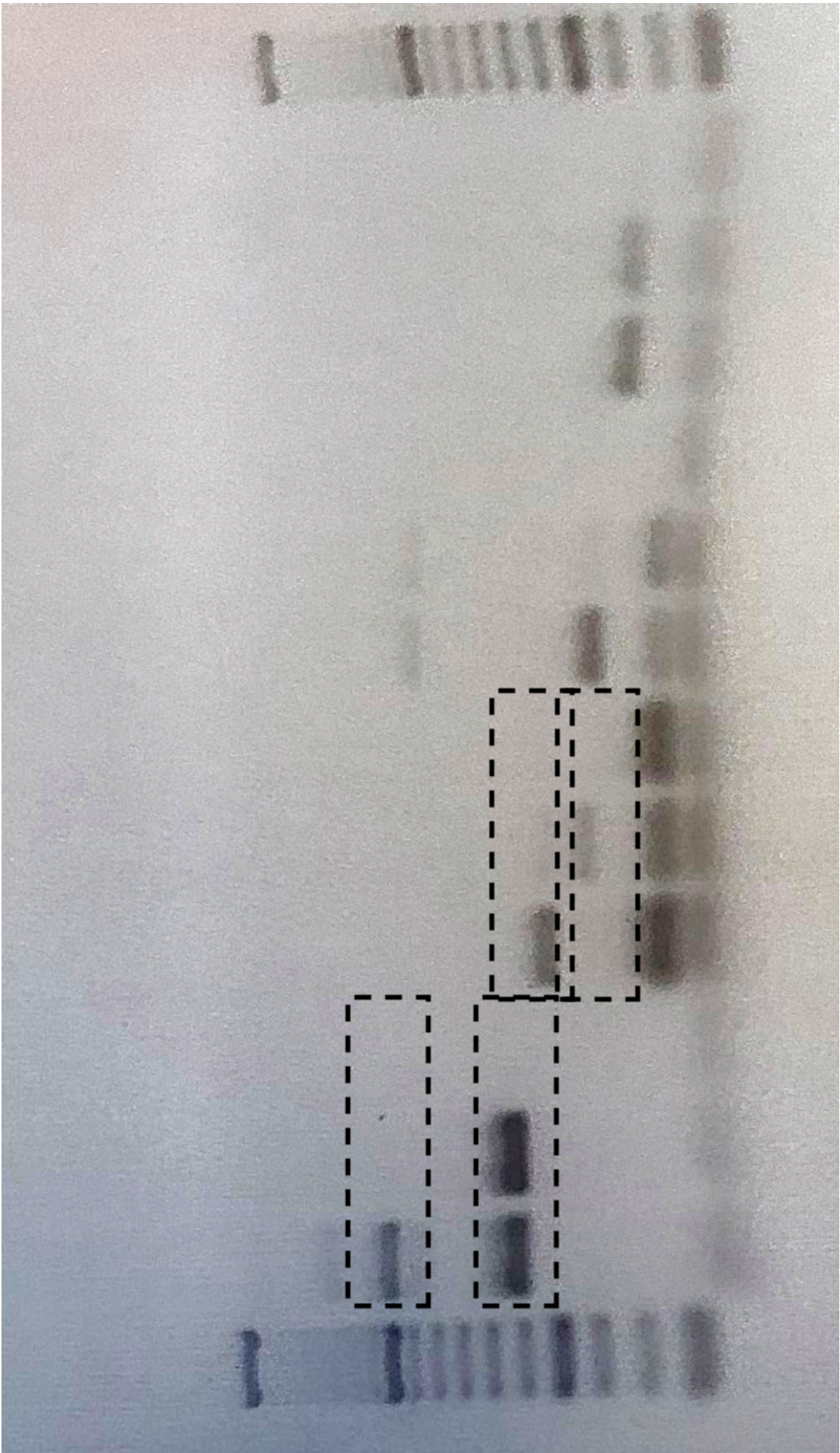

Supplement: Supplementary file 7 — Unprocessed gels for Fig. 1. [file 41594_2025_1565_MOESM7_ESM.pdf]
